# Classifying Germinal Center Derived Lymphomas—Navigate a Complex Transcriptional Landscape

**DOI:** 10.3390/cancers14143434

**Published:** 2022-07-14

**Authors:** Henry Loeffler-Wirth, Markus Kreuz, Maria Schmidt, German Ott, Reiner Siebert, Hans Binder

**Affiliations:** 1 Interdisciplinary Centre for Bioinformatics, University Leipzig (IZBI), 04107 Leipzig, Germany; wirth@izbi.uni-leipzig.de (H.L.-W.); schmidt@izbi.uni-leipzig.de (M.S.); 2 Fraunhofer Institute for Cell Therapy and Immunology (IZI), 04103 Leipzig, Germany; markus.kreuz@izi.fraunhofer.de; 3 Department of Clinical Pathology, Robert-Bosch-Krankenhaus, Dr. Margarete Fischer-Bosch Institute of Clinical Pharmacology, 70376 Stuttgart, Germany; german.ott@rbk.de; 4 Institute of Human Genetics, Ulm University and Ulm University Medical Center, 89073 Ulm, Germany; reiner.siebert@uni-ulm.de

**Keywords:** molecular classifiers, gene expression, dark and light zone functions, tumor heterogeneity, subtyping, marker sets

## Abstract

**Simple Summary:**

Germinal center-derived B-cell lymphomas constitute a very heterogeneous group of neoplasms with diverse clinical presentations, prognoses, and responses to therapy. They divide into a series of subtypes, such as Diffuse Large B-cell lymphomas (DLBCL), Burkitt Lymphomas (BL), Follicular lymphomas (FL), and further, into several subclasses and rarer subtypes of finer granularity, which makes them one of the most heterogeneous cancer entities. Molecular classification schemes, first of all, derived from whole transcriptome gene expression data largely improved subtyping and functional understanding. Based on a whole transcriptome landscape of B-cell lymphoma, we show that one major caveat in the task of classification is represented by the rather fuzzy distribution of individual tumors without clear-cut borderlines between most of the subtypes, preventing their unambiguous association with clear-cut entities. This landscape is governed by the germinal center (GC) reaction and relates different subtypes to different states along the reaction path. We discuss the relatedness between the expression landscape and classifier signatures and their functional cell of origin background. This view helps to stratify lymphomas in terms of modular building blocks of signature genes and to interpret rarer subclasses in the context of larger ones.

**Abstract:**

Classification of lymphoid neoplasms is based mainly on histologic, immunologic, and (rarer) genetic features. It has been supplemented by gene expression profiling (GEP) in the last decade. Despite the considerable success, particularly in associating lymphoma subtypes with specific transcriptional programs and classifier signatures of up- or downregulated genes, competing molecular classifiers were often proposed in the literature by different groups for the same classification tasks to distinguish, e.g., BL versus DLBCL or different DLBCL subtypes. Moreover, rarer sub-entities such as MYC and BCL2 “double hit lymphomas” (DHL), IRF4-rearranged large cell lymphoma (IRF4-LCL), and Burkitt-like lymphomas with 11q aberration pattern (mnBLL-11q) attracted interest while their relatedness regarding the major classes is still unclear in many respects. We explored the transcriptional landscape of 873 lymphomas referring to a wide spectrum of subtypes by applying self-organizing maps (SOM) machine learning. The landscape reveals a continuum of transcriptional states activated in the different subtypes without clear-cut borderlines between them and preventing their unambiguous classification. These states show striking parallels with single cell gene expression of the active germinal center (GC), which is characterized by the cyclic progression of B-cells. The expression patterns along the GC trajectory are discriminative for distinguishing different lymphoma subtypes. We show that the rare subtypes take intermediate positions between BL, DLBCL, and FL as considered by the 5th edition of the WHO classification of haemato-lymphoid tumors in 2022. Classifier gene signatures extracted from these states as modules of coregulated genes are competitive with literature classifiers. They provide functional-defined classifiers with the option of consenting redundant classifiers from the literature. We discuss alternative classification schemes of different granularity and functional impact as possible avenues toward personalization and improved diagnostics of GC-derived lymphomas.

## 1. Introduction

Lymphomas and other cancers are collections of genetic diseases with different pathological, molecular-functional and clinical outcomes even for neoplasms of the same primary site of genesis and growth. The revised 4th Edition of the World Health Organization (WHO, see glossary below) classification of lymphoid neoplasms defined 51 mature B-cell neoplasm entities [1,2], thus representing probably one of the most heterogeneous tumor families. Classification of lymphoid neoplasms is based mainly on histologic, immunologic, and (rarer) genetic features. In recent years, the diagnosis of lymphomas has been supplemented by molecular classification schemes, initially based on gene expression profiling (GEP) [3,4,5,6,7,8,9,10] followed by mutational patterns [11,12] and also by combinations of both [13,14,15]. Diffuse large B-cell lymphoma (DLBCL) is the most frequent subtype of mature B-cell lymphomas accounting for around 40% of all lymphomas and features extreme molecular and clinical heterogeneity. The cell-of-origin (COO) classification [16] comprises presently the most common subtyping approach of DLBCL reflecting its developmental stage in the germinal center (GC). It distinguishes germinal center B-cell-like (GCB) and activated B-cell-like (ABC) subgroups as well as a grey-zone, “unclassifiable” (also named Type III) category [17,18]. In addition, a series of alternative models employed GEP for subtyping DLBC based on different molecular contexts such as energy metabolism, proliferation, inflammation [7,9,10,19], tumor microenvironment [20], B-cell phenotypes [21] or distinct pathway activation patterns [3,8] (see also [22] for a review). B-cell lymphoma entities the GEPs of which partially overlap with DLBCL are Burkitt lymphoma (BL) and Follicular lymphoma (FL) as well as related (provisional) entities of “intermediate” character between BL and DLBCL (see below). Novel schemes account for recognition of the continuous nature of transcriptional state space by defining subtypes at a finer granularity scale extracted from the diversity of tumor expression patterns [4] or decomposition of their independent components [23] or from GC-cell states identified by means of single-cell transcriptomics [24,25]. “Now, after 20 years, COO testing for DLBCLs has lost its luster but is not yet obsolete despite its questionable prognostic value and lack of predictive utility” having been recently stated [26].

Molecular classification schemes can utilize single marker genes or sets of them as classifiers whose mRNA levels in tumor samples enable one to predict their class membership after GEP. Only a few of these molecular features, however, have yet been considered in the WHO classification of lymphoid neoplasms [1]. This is presumably due to the lack of available consensus technologies and clinical studies, but also, and probably first of all, to the existence of a series of competing molecular classifiers proposed independently by different groups for the same classification task often without mutual reference and comparison. Users are then left with the problem to choose between classifiers because benchmarking information or contextual details are scarce or even lacking. In our previous studies of lymphomas [4] but also of other tumor entities such as melanomas [27], gliomas [28] and colon cancer [29], we found that independent sets of marker genes published by different authors perform in a similar fashion in our data even if the respective genes sets weakly overlap. Hence, multiple seemingly different classifiers often pop up in the literature for the very same question which obviously reflects a widely distributed redundancy regarding diverging sets of marker genes usually with unclear differences in their prediction accuracy and the underlying biological context.

This controversial issue with several competing classifiers directly relates to the multidimensional and complex nature of the underlying data landscape of lymphomas. We recently generated such a holistic transcriptional landscape of lymphomas by applying self-organizing maps (SOM) machine learning to a data set of about 900 lymphomas referring to a wide spectrum of subtypes [4]. In this publication, we aim at evaluating the stability and interpretability of different sets of lymphoma markers taken from the literature [3,5,17,18,30,31] in the context of this landscape. In the first part of the paper, we describe details of this landscape and their biological meanings in terms of subtype-specific transcriptomic portraits for DLBCL, BL and FL as well as for rarer entities such as aggressive lymphoma with simultaneous rearrangements of MYC and BCL2 (so called “double hit lymphomas”; DHL), *IRF4*-rearranged large cell lymphoma (*IRF4*-LCL), and *MYC*-negative high grade B-cell lymphomas (formerly called Burkitt-like lymphomas) with 11q aberration pattern (mnBLL-11q). We also consider the split of DLBCL into ABC- and GCB-types related to GC-development and discuss footprints of the normal GC-reaction in the lymphoma landscape. In the second part, we extract classifier gene sets for the different lymphoma subtypes (BL, DLBCL, ABC, GCB, DHL) from the expression landscape and compare them with classifiers from the literature. In the last part, we discuss possible avenues to further develop classification of lymphomas.

## 2. Material and Methods

### 2.1. Lymphoma Data

We here reanalyzed microarray-based expression values of 873 biopsy specimens of mature B-cell lymphomas and of 40 reference samples (tumor cell lines, sorted B-cells, tonsils). Reference samples were not explicitly addressed in this publication; please see [4] for details, particularly Supplementary Material, Table S1 in [4]. The lymphoma samples divide into ten major strata: (i) diffuse large B-cell lymphoma (DLBCL, 430 cases), (ii) follicular lymphoma (FL, 145 cases), (iii) “intermediate” lymphoma according to [3] (81 cases), (iv) prototypic Burkitt lymphoma (BL, 74 cases), (v) mixed FL/DLBCL and WHO grade 3B FL (48 cases), (vi) mediastinal large B-cell lymphoma (PMBL, 23 cases), (vii) multiple myeloma (MM, 20 cases), (viii) *IRF4*-rearranged large cell lymphoma (*IRF4*-LCL, 10 cases), (ix) *MYC*-negative Burkitt-like lymphomas with 11q aberration (mnBLL-11q, 6 cases), and (x) mantle cell lymphoma (MCL, 4 cases). DLBCL were further stratified into the cell of origin (COO) groups, germinal center B-cell like (GCB, 142 cases), activated B-cell-like (ABC, 133 cases), and unclassified (97 cases) DLBCL and DHL (58 cases) lymphomas. Cases have been classified according to the 4th Edition of the WHO classification, but Table 1 provides a translation into the 5th Edition [32].

### 2.2. Transcriptome Map of Lymphomas and Spot Classifiers

The expression data were used to generate a self-organizing map (SOM) which distributes the genes under study in a 50 × 50-pixel grid such that co-expressed genes across the samples are clustered together in the same or in adjacent pixels called metagenes. The SOM then provides “portraits” of all individual samples and, after group-averaging, of the different lymphoma subtypes by color coding the expression level of the metagenes. Co-expressed, high variant metagenes cluster together according to the self-organizing properties of the SOM forming so-called spot-like areas in the portraits. They were used to extract sets of marker genes as potential classifiers for subtypes showing specific overexpression of the respective spot. Spot genes were selected as described previously [4].

### 2.3. Reference Classifiers, Gene Set Maps and GSZ-Profiles

We studied reference classifiers taken from six publications developed to discriminate BL-vs-DLBCL, ABC-vs-GCB DLBCL and DHL-vs-GCB DLBCL, respectively [3,5,17,18,30,31] (see Table 2 below). Notably, some of these reference classifiers have been derived and/or validated from data of other quoted classifiers, thus, not all of them can be regarded as independent. The classifiers comprise signature sets of genes, which have been obtained from two-class comparisons based on different lymphoma test data as specified in Table 2. For the sake of simplicity and mutual comparisons, we processed classifier signatures in a standardized way as follows: For each two-group comparison, classifier genes were split into two subsets either upregulated in the one or in the other group of lymphomas: e.g., for BL-versus-DLBCL classification, the signature comprises two sets of genes overexpressed either in BL (BL_up alias DLBCL_down) or DLBCL (DLBCL_up alias BL_down), respectively. We explicitly acknowledge that this way of processing dismisses any information from weighting of single gene expressions. The functional context of the classifier genes was estimated by mapping them into the SOM of the MMML data set (Molecular Mechanisms of Malignant Lymphomas consortium, see [4] for the detailed description). The obtained gene set maps visualize the location of all genes of the reference signatures in the SOM. Their accumulation in or near certain spots reflects co-expression and possible functional association. Each (reference or spot) classifier comprises a set of genes (alias signature). It provides one GSZ (gene set Z-score)-value per lymphoma case which estimates its expression level in this particular sample. GSZ values of the signatures were used for classification and calculation of receiver operator curves (ROC). Additional materials and methods descriptions are provided in Appendix A regarding lymphoma data (Appendix A.1), GSZ Profiles of Classifier Signatures and ROC Characteristics (Appendix A.2), an interactive oposSOM Browser of the MMML Lymphoma Data Set available online (Appendix A.3) and an application of Machine Learning of Transcriptonal Portraits to the data (Appendix A.4).

## 3. Results

### 3.1. The Multi-Dimensional Nature of the Lymphoma Transcriptome

**Figure 1:**
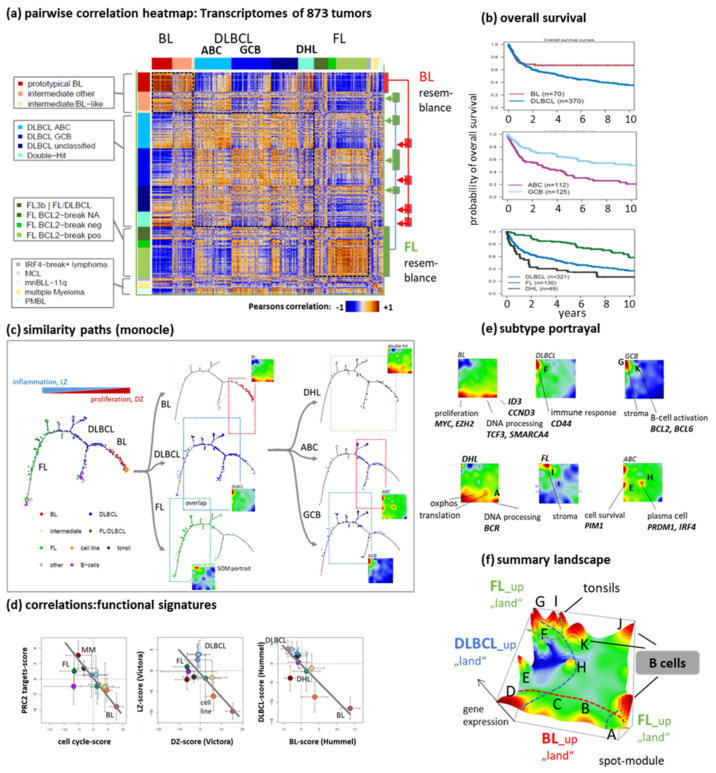
The complex transcriptional landscape of lymphomas: (**a**) The pairwise correlation map reveals a high diversity of similarities (red) and dissimilarities (blue) between different lymphoma strata. The color-code of lymphoma classes is used throughout the paper. (**b**) Overall survival curves reveal different prognoses for the subtypes, which makes diagnostics an important task (survival data were taken from [4]). (**c**) Similarity tree presentations illustrate the distribution of major lymphoma subtypes. They reflect rather a continuum of molecular states than well-separated clusters of lymphoma subtypes. Subtypes considerably overlap (as visualized by the rectangles), which challenges classification based on the gene expression data. A similarity tree was generated using “monocle” [36]. (**d**) Correlation plots show virtually alternate changes of the mean expression scores of different functional context, namely, of targets of the PRC2 (with impact for epigenetic dedifferentiation of cellular programs [37]) as a function of cell-cycle activity; of LZ-versus-DZ activity scores [38] and of DLBCL-versus-BL classification signatures [3]. The grey line serves as a visual guide. (**e**) Transcriptome SOM portraits of selected subtypes obtained from the MMML-cohort show specific expression patterns where red overexpression “spot”-modules are associated with molecular function and key genes (see [4] for details, spots are labelled with capital letters). (**f**) The spot summary map provides an overview about the overexpression spot modules observed in lymphomas (see part e). Spots are assigned with capital letters. Their expression profiles are shown in Figure A3. Spots upregulated in BL, DLBCL and FL are found in distinct areas, “lands” of the landscape which partly overlap (dashed borderlines). “B-cell” functions refer to pre- and post-GC B-cells as well as activated genes of the B-cell receptor pathway (see [4] for details).

To study the diversity of B-cell lymphomas, we applied SOM portrayal to microarray gene expression data of 873 biopsy specimens referring to a large spectrum of lymphoma subtypes collected within the framework of the MMML (see [4] and materials and methods section for details). We utilized the sample portraits to build a pairwise correlation heatmap illustrating mutual similarities (red) and dissimilarity (blue) relations (Figure 1a). One finds roughly three larger groups of BL-like, DLBCL-like and FL-like tumors along the diagonal as red correlation clusters but also a high degree of heterogeneity in terms of red off-diagonal stripes particularly indicating partial BL- or FL-resemblance of part of the DLBCL. Though the clinical data have to been interpreted with caution in this multi-center retrospective of mostly pre-Rituximab series, the major subtypes differ in prognosis and the shapes of the survival curves underlining the need for practicable and robust classification schemes (Figure 1b).

Next, the heatmap was transformed into a similarity tree to visualize the relatedness between the major tumor entities (Figure 1c). FL, DLBCL and BL occupy certain regions along the tree, which, however, considerably overlap. Similar results were obtained for GCB- and ABC-type DLBCL and DHL (Figure 1c, right part). The tumors form a continuum of transcriptional states without clear-cut borderlines between the different strata. Therefore, any sharp delineation of subtypes based on GEP without overlapping grey zones will always be challenging, if not impossible.

Correlation plots of the mean expression of the lymphoma subtypes between functional gene set scores of an epigenetic signature (targets of the polycomb repressive complex 2, PRC2) and cell cycle activity as well as between activity scores of the light zone (LZ) and dark zone (DZ) of the GC are roughly linear between DLBCL (and FL) on the left and BL on the right (Figure 1d). The choice of these characteristics was motivated by recent studies which revealed an antagonistic regulation between proliferative and inflammatory programs in BL- and DLBCL- (and partly also FL-) like lymphomas, respectively. It associates with the activation of *MYC*-targets and open chromatin states on one hand and with the activation of targets of the repressive PRC2-complex, aberrant DNA-methylation, repressed chromatin states as well as the reprogramming of the machinery of writers and erasers of epigenetic marks on the other one [33,34].

A classifier signature set obtained by means of BL-versus-DLBCL differential expression analysis [3] shows a similar dependence as a DZ-versus-LZ origin would imply (Figure 1d, right plot). These virtually one-dimensional changes, however, oversimplify the real picture of transcriptional covariations. The multidimensional nature can be visualized in terms of transcriptomic “portraits” of the subtypes (Figure 1e). The portraits reveal modules of co-expressed genes by means of red (upregulated) and blue (downregulated) “spot”-like clusters labeled by capital letters for assignment (Figure 1e, modules are also called “spots’ in the following). The activated modules in the portraits provide fingerprint patterns of the respective transcriptional state. In BL, activated modules enrich genes related to proliferation (spot D) and DNA processing (spot B); DLBCL display higher expression of genes which are associated with immune response, inflammation and extracellular matrix (spot F and G), and FL have increased expression of genes of stromal characteristics [35] (spot I). ABC-DLBCL specifically activate genes in spot H related to plasma cell maturation, also activated in MM [4]. The different spots contain marker genes upregulated in the respective subtypes such as *MYC* (BL and part of DLBCL, spot D), *ID3* and *TCF3* (BL, spot B) or *PRDM1* and *IRF4* (ABC-DLBCL, spot H). Expression profiles of the spot modules are shown in Figure A3 and accumulation of functional signatures in and around the spots in Figure A4. Note that the scores used in the bi-plots in Figure 1d can be substituted by functional signatures accumulating in spots D and F such “*MYC*-targets” or “stroma”, respectively, without changing the plots significantly. The spot-landscape can be summarized as overexpression summary map which divides into “lands” containing spots upregulated in the different subtypes (Figure 1f).

In summary, lymphomas distribute in a complex transcriptomic similarity landscape. Subtype-related portraits show clear differences which associate with specific biological functions. One major caveat in the task of classification, however, is represented by the rather fuzzy distribution of individual cases without clear-cut borderlines between most of the subtypes preventing their unambiguous association with clear-cut entities.

### 3.2. Beyond the Canonical DZ-LZ Dualism

**Figure 2:**
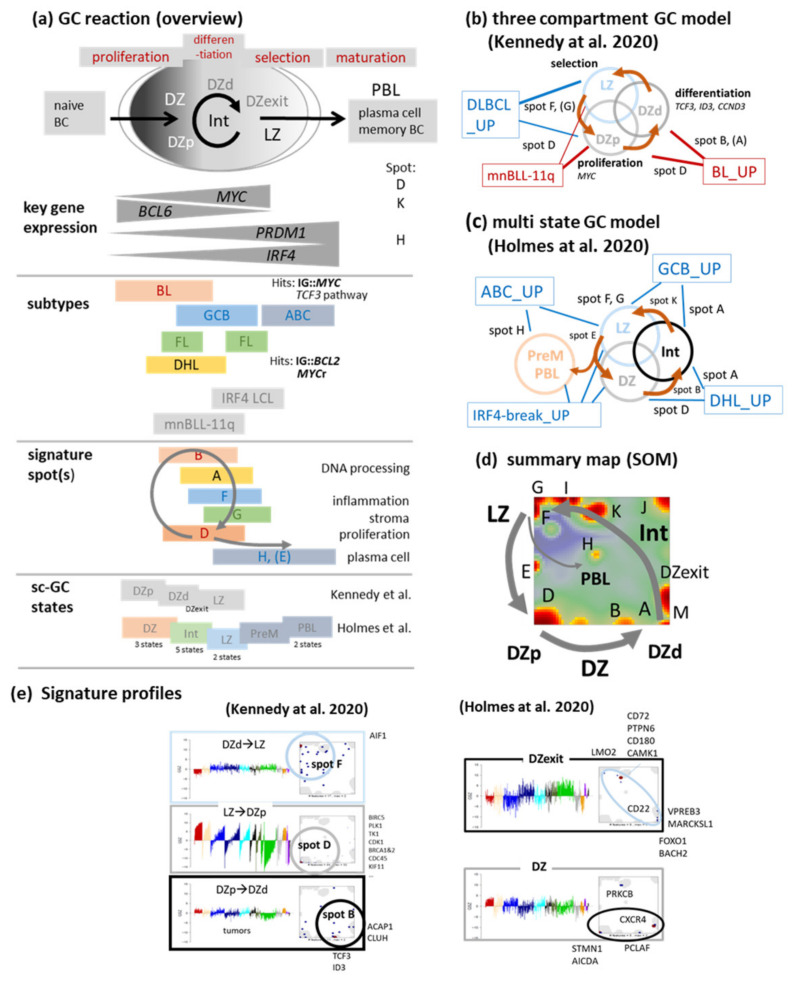
Beyond the GC-bipartition as seen by single-cell transcriptomics: (**a**) Schematic view of the GC reaction and its relation to spot expression. Naïve (pre-GC) B-cells maturate upon passing the germinal center (GC) into maturated B-cells and plasma cells. The GC divides into the dark (DZ) and light (LZ) zones, which associate with different functions and microenvironments, with typical expression changes of marker genes, spot-modules and lymphoma subtypes (adapted from [41,50], see also [42]). (**b**) Kennedy et al. [25] identified two separated cell states in the DZ fulfilling proliferative (DZp) and differentiating (DZd) functions, which tripartite the GC into DZp, DZd and LZ compartments. Gene signatures of them associate with spots D and B (and A), respectively (see part (**e**)). BL and *MYC*-activated DLBCL share activated spot D while FL, DHL and BL share activation of spot A. Spot B is uniquely activated in BL (see profiles in Figure 1e). (**c**) The multistate model divides the GC reaction into DZ-, INT- (intermediate), LZ- and PBL-states (see Figure 3 and [24]). (**d**) The GC reaction appears as closed circle in the overexpression summary map visualizing the expression state space of lymphomas. It links spots G/F, D, B, A and K while plasma cell maturation forms a side branch towards spot H. (**e**) Signatures of GC states taken from both publications accumulate in distinct areas of our transcriptome SOM associating the respective spots with the GC reaction (Figure A5 and Figure A6 for more details). DZexit was identified as expression state preparing the cells to exit DZ towards LZ via INT [24]. Tumors are ranked with increased cell cycle activity for each subtype. Spot D reflects strong association with cell cycle activity (ramp-like silhouettes) while spot B shows not (see Figure A3).

In a simplified view, GC B-cells divide into two canonical populations, showing either dark zone (DZ) or light zone (LZ) phenotypes [38,39,40]. Within the active GC, orchestrated molecular programs must balance proliferation and selection to provide effective humoral immunity and to protect against genomic instability and neoplastic transformation (Figure 2a). The GC reaction is based on complex spatial and temporal dynamics that are still only partially elucidated. The GC is also the site in which most mature B cell lymphomas originate [41], suggesting that the process of malignant transformation disturbs the physiology of GC and post-GC differentiation by stalling lymphoma cells at various stages of the GC reaction [42] and/or de-synchronizing their transcriptional programs [43]. For example, BL and DLBCL on the average show high and low DZ scores, respectively, which associate with proliferative activity and epigenetic re-programming, respectively (Figure 1d) [37,41,44].

Recent single-cell transcriptome studies provided a more detailed view, segregating the GC into more than the two morphologically distinguishable compartments [24,25,43,45,46]. Of note, two distinct B-cell populations have been described by Kennedy et al. [25,47] in the DZ that differ both with respect to function and location: Proliferating DZ (DZp) cells transform into differentiating DZ (DZd) cells before they exit the DZ and re-enter the LZ (Figure 2b, [25,47]). Interestingly, gene signatures of the three compartments taken from [25] are found in/near spot areas F (LZ_up), D (DZp_up), and B (DZd_up) (Figure 2e and Figure A5), suggesting that our transcriptomic state space well recapitulates this tri-partition of the GC (Figure 2d). Key genes of these compartments, namely, *MYC* and *TCF3*, locate in different spot modules D and B, respectively. The former is a driver of proliferation in DZp while the latter acts as primer for *E2A* targets in DZd [25]. *MYC* is a global cancer driver deregulated in virtually all BL (typically, via an *IG*::*MYC* translocation) but also in roughly 30–40% of DLBCL featuring increased proliferation-associated transcription as reflected by upregulated expression of spot D (Figure 2d, see also [48] and references cited therein). Genes of the *TCF3* pathway (*TCF3*, *ID3*, *CCND3*) constitute targets of BL-specific second mutation hits in around 90% of BL, following *MYC*-translocation [49] rendering BL differently from *MYC*-translocated DLBCL. These three *TCF3*-pathway genes locate in or near spot B which specifically upregulates in BL due to second hits and thus possibly reflects stalling of BL in the DZd (and/or DZp) compartment(s) (Figure A5).

**Figure 3:**
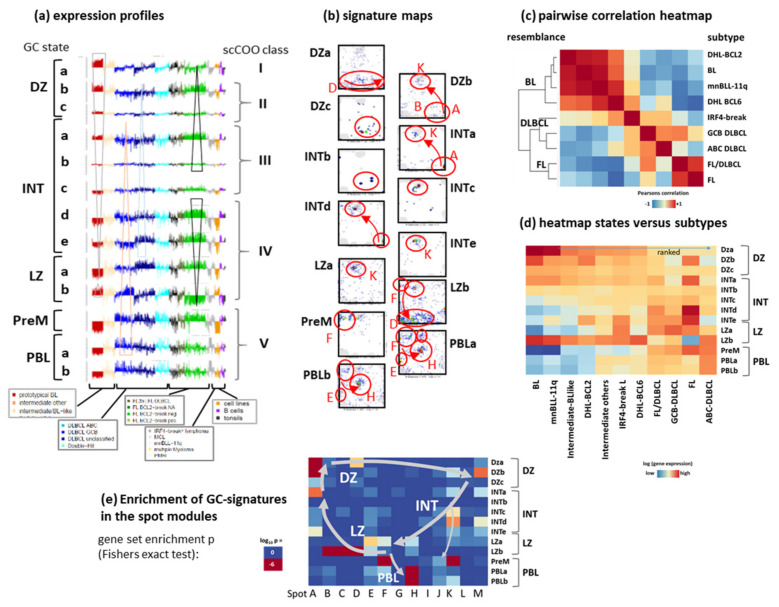
Gene expression signatures of the multistate model of Holmes et al. [24]: (**a**) GSZ-profiles of the 13 GC cell states across the lymphoma tumors. It divides into transcriptional states of GC B-cells distributing across the LZ, DZ and intermediate (INT) GC compartments and in addition, states referring to precursor memory (PreM) and plasmablast (PBL) cells, both showing commitment towards memory and plasma cells, respectively. Tumors of each subtype were ranked with increasing cell cycle activity from the left to the right. scGC signatures were taken from [24] (Table S2 there, UP signatures). (**b**) Maps of the signature genes of the scGC-states. Areas of accumulation are shown by ellipses. The GC reaction can be summarized into a closed circle in the SOM (see arrows and Figure 2). (**c**) The heatmap visualizes mutual similarities between the mean SOM expression portraits of different lymphoma strata. The most prominent cluster collects BL- and FL-resembling subtypes with DLBCL-like subtypes in between. (**d**) Clustered heatmap of the mean expression of the GC-states versus the lymphoma subtypes. (**e**) Enrichment of GC-state signature sets in the spot modules (log *p* values, Fishers exact test). Spots with enriched signatures (brown areas in the heatmap) form the GC-reaction circle shown in the landscape in Figure 2d; see also Figure A6.

Another single-cell transcriptomic study identified thirteen GC B-cell states which distribute over the DZ, LZ, an intermediate phenotype of B-cells (INT), and include also B-cells that just exit the DZ (DZexit), and in addition, precursors of memory B-cells (PreM) and plasmablasts (PBL) [24] (Figure 2c). We considered signatures of the thirteen states referring to DZ (DZa-c), INT (INTa-d), LZ (LZa,b) as well as PreM and PLB (PLBa,b) as provided in [24] and analyzed their GSZ profiles, distribution in the SOM landscape, mean expression across the lymphoma subtypes as well their accumulation in the different spots (Figure 3). Key genes of PBL such as *PRDM1* and *IRF4* locate in spot H in our landscape upregulated in ABC-DLBCL (Figure 1e) and MM [4]. Signature genes of the DZ, INT and DZexit states are found in spots D, B and A, K, respectively (Figure 2d,e and Figure 3b,e). Overall, the transcriptomic states of these different GC B-cell phenotypes form a closed trajectory in the expression state space as provided by the spot summary map. It reflects B-cell development in the GC by connecting LZ (spot K and F), proliferative DZp (spot D), developmental DZd (spot B and A), and INT (spot A and K) expression states, while PreM and PBL (spot H) form a side branch featuring plasma cell maturation (Figure 2d and Figure 3). This side branch links with the final LZb state, combining expression characteristics of DZa (spot D, proliferation), LZa (inflammation, spot F) and PBL (spot H, Figure 3). Notably, this trajectory is not characterized by the strict serial activation of the spots but mostly by the combinatorial activation of two to four spots along the trajectory in each of the GC-states (Figure 3a,b,e) in correspondence with our previous pattern-type (PAT) analytics of lymphoma heterogeneity [4] (see Figure A6b).

In summary, recent GC B-cell single-cell transcriptomics in different settings revealed several DZ B-cell types such as the DZ, DZexit, partly intermediate [24], DZp and DZd [25], which all group along a closed ‘GC reaction trajectory’ in the expression landscape. The expression profiles of the spots along the GC trajectory are discriminative for distinguishing different lymphoma subtypes (Figure 2e) and partly resemble signatures of GC B-cell populations in agreement with a cell-of-origin classification derived from single-cell expression phenotypes [24]. Overall, these results provide indications for the cyclic progression of B cells in the GC which extends the complexity of the GC reaction beyond the DZ–LZ dualism with possible impact on lymphoma subtype specifics. Of note, the gene expression signatures associated with the cell subpopulations of GC B-cells provided sc-COO classifiers for novel—putatively prognostic—subgroups of DLBCL with relation to LZ (light zone) and DZ (dark zone) GC functions [24,25].

### 3.3. Provisonal Genetic Entities: mnBLL-11q, IRF4-Break-LCL and HGBL-DH

Subtyping schemes do not only pursue the major goal of describing recognized and reproducibly diagnosable entities but also aim at the recognition of putative novel categories that require further studies: High-grade B-cell lymphomas with MYC and BCL2 or BCL6 (double/triple hit) rearrangements (HGBL-DH/TH), High-grade B-cell lymphomas, not otherwise specified (HGBL, NOS), Large B-cell lymphoma with IRF4 rearrangement (IRF4-LCL) and high grade B-cell (previously Burkitt-like) lymphoma with 11q aberrations (mn-BLL11q) were introduced, still awaiting their definite positioning in the molecular landscape of lymphomas [4,5,6,30,51,52,53]. The pairwise correlation heatmap of the expression portraits of these entities indicates the relatedness between them (Figure 3c). One observes a cluster of BL-resemblance which includes DHL-*BCL2* and DHL-*BCL6*, mnBLL-11q, and partly, *IRF4*-breakL, as well as a DLBCL-like and an FL-like cluster with a certain overlap between them reflecting a transition range between DLBCL and transformed FL [54,55]. The expression of the subtypes across the GC states further refines the relatedness between them (Figure 3d). BL, intermediate L, mn-BLL11q and DHL form a DZ-like cluster, GCB-DLBCL and FL/DLBCL are more LZ-like while FL distributes mostly across INT and LZ states, IRF4-break LCL are bimodally distributed between DZ and LZ (and partly, PBLb) and ABC-DLBCL show PBL properties. Note also that the proliferative late LZb state resembles DZ-like expression except in ABC-DLBCL, which supports the view that the paths of re-entry into DZ and towards plasma blasts split in the LZb compartment.

**Figure 4:**
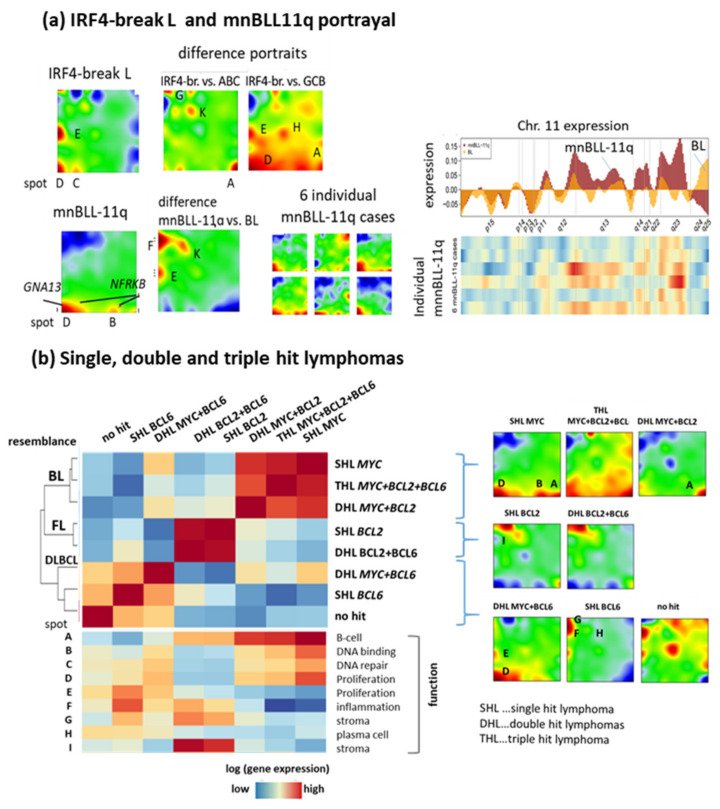
Provisional “intermediate” lymphoma types. (**a**) The portraits of *IRF4*-breakL and of mnBLL-11q were complemented by difference portraits with respect to DLBCL and BL, respectively, which indicate transcriptional differences. mnBLL-11q are characterized by overexpression along the Chr. 11q arm. (**b**) Comparison of single hit lymphomas (SHL-MYC, -*BCL2*, -*BCL6*) with respective double hit (DHL) and triple hit (THL) regarding pairwise similarities, spot expression and SOM portraits. BL-resembling classes activate spots A–D, FL-like spot I.

Transcriptomic portraits of *IRF4*-break LCL and mnBLL-11q partly resemble the portraits of ABC-DLBCL and BL, respectively (Figure 4a). Difference portraits between *IRF4*-breakL and ABC- as well as GCB-DLBCL reveal increased levels of spot D (proliferation) and E (*PIM1*) and decreased levels of spot H (*PRDM1*, *IRF4*) and F (inflammation) compared with their *IRF4* break negative counterparts in agreement with [51]. A model of “kinetic control” [56] suggests that IRF4 regulates immunoglobulin class switch recombination and plasma cell differentiation by mutually antagonistic cell fate decisions into B- and plasma-cell transcriptional programs. mnBL11q feature DLBCL signatures such as elevated expression of spots E (*PIM1*), F (inflammation) and K (B-cell activation, *BCL6*), reflecting intermediate characteristics between BL and DLBCL. Detailed inspection of gene expression along chromosome 11 revealed marked activation along the q-arm associating with chromosomal gains [57], however, with considerable variation between individual cases (Figure 4a). Hence, consideration of specific genetic lesions increases granularity of subtyping schemes towards the “intermediate” range between DLBCL and BL. Details of the molecular mechanisms leading to “BL-resemblance” remain, however, unclear in many details.

Consideration of transcriptome changes along the GC reaction provides a few insights into molecular mechanisms of mnBLL-11q. Strikingly, these *MYC*-negative lymphomas resemble BL in transcriptional features but lack recurrent mutations in the *ID3-TCF3* axis [58,59] (see above). However, *GNA13*, a gene mutated in around 25% of BL and also in GCB-DLBCL ([60] and references cited therein) is mutated in 50% mnBLL-11q. *GNA13* mutated GC B-cells seem to persist in the GC B differentiation state because of uncoupling between affinity selection and cell survival. Another gene recurrently mutated in mnBLL-11q, *NFRKB*, locates at Chr.11q24, a region of chromosomal deletions and downregulated expression compared with BL (Figure 4a) which presumably affects transcriptional regulation by chromatin remodeling as a cofactor in the *INO80* complex in parallel and in analogy to SWI/SNF, a chromatin remodeler frequently deregulated in BL [58]. Both *GNA13* and *NFKRB* genes locate in/near spots D and B activated in mnBLL-11q, however, at a lower level than in BL (compare main, individual, and difference portraits in Figure 4a). The difference portrait of mnBLL-11q compared with BL also indicates an increased expression of spot F (inflammation) reflecting a slight shift towards LZ resemblance, and thus, towards DLBCL and/or high-grade B-cell lymphomas [59]. Hence, mnBLL-11q seem to be locked in a DZp resembling transcriptional state in partial analogy to BL, which, however, is driven by different (epi-)genetic determinants and shows slightly enhanced inflammatory, LZ-resembling characteristics in partial analogy with highly proliferative DLBCL cases.

HGBL-DH/TH (high grade B-cell lymphoma with double/triple hits) harbor simultaneous translocations of *MYC* and *BCL2* (DHL *BCL2*) or *MYC* and *BCL6* (DHL *BCL6*), which associate with aggressive clinical course and inferior prognosis [6,61]. Comparison of the mean SOM expression portraits revealed that tumors classified as BL, mnBLL-11q and DHL *BCL2* form a cluster of closest BL resemblance while DHL *BCL6*, although also similar, form a distinct side branch (Figure 3c). For a more detailed view, we compared the expression portraits of the different types of DH/TH and single hit lymphomas (SHL, Figure 4b). SHL *MYC*, DHL *BCL2* and THL form a cluster of BL resemblance overexpressing, first of all, spot A, while the other entities distribute over the FL- and DLBCL-like clusters. DHL *BCL2* contrast with DHL *BCL6* in the overexpression of spot A and under-expression of spot F (inflammation) while both HGBL types show enhanced proliferative activity (spot D), however, distinctly lower compared with BL and mnBLL-11q (Figure 4a,b). In HGBL, the presence of a *MYC* translocation seems to dominate the resulting phenotype conferring BL resemblance. The marked singular overexpression of spot A specifically characterizes DHL *BCL2* and THL but it is also observed in BL (see portraits in Figure 4b) and, to a lesser degree, in FL, thus constituting an “overlap” expression feature of BL and FL. Overexpression of spot A suggests that DHL *BCL2* combine dark zone and especially DZd and partly DZexit features of the GC reaction with FL characteristics, while expression of DHL *BCL6* is more similar to the proliferative DZp state (Figure 3d). Hence, these lymphoid tumor types take distinct intermediate positions in between the core subtypes caused by specific genetic lesions. The 5th edition of the WHO classification of haemato-lymphoid tumors 2022 acknowledges these similarity relations between the lymphoma entities and segregates DH *BCL6* as a subtype of DLBCL, NOS or HGBL, NOS apart from the DH *BCL2* (Table 1).

### 3.4. Gene Expression Classifiers of BL and DLBCL

**Figure 5:**
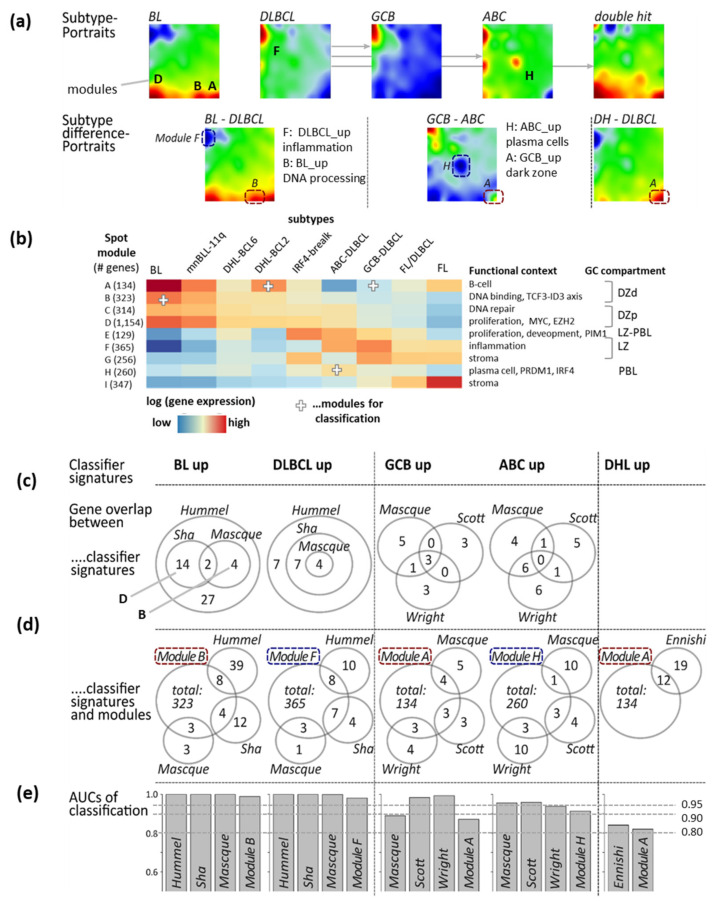
SOM portrayal and subtype classifiers of B-cell lymphomas: (**a**) SOM portraits of selected B-cell lymphoma subtypes (BL, DLBCL, ABC-, GCB- DLBCL and DHL) show specific expression patterns characterized by “spot” modules of overexpressed genes (red spots, labeled by capital letters in agreement with [4]). Difference portraits reveal differentially expressed genes in red (up) or blue (down). (**b**) The heatmap provides an overview of spot expression in the different subtypes together with major functional context and assignment to compartments of the GC reaction. White crosses indicate spots used for classifiers in this publication. Full lists of spot genes are provided in the Supplementary Material of [4]. (**c**) Different classifier gene sets for two-group comparisons (BL-vs-DLBCL, ABC-vs-GCB, DHL-vs-nonDHL) taken from literature often only moderately mutually overlap due to selection uncertainty and function bias of the test data used for classifier “building” (Table 2). The confusion table of jointly detected samples by applying the different classifiers is provided in Figure A7. (**d**) Overlap of literature classifiers with spot modules. (**e**) Literature classifiers almost similarly perform in terms of area under (ROC) curve (AUC) values. Although spot modules were not specially designed for classifying tasks, they provide almost similar results for discriminating subtypes.

The different lymphoma subtypes show distinct SOM expression portraits which are characterized by specific activation patterns of one or more spot-modules of co-expressed genes. In the next step of our analysis, we asked how the genes from these spots perform as classifiers for a certain subtype, how they compare with GEP classifier signatures selected from the literature, how the literature classifiers map into the SOM expression landscape, and how they fit into the functional context of this landscape, particularly with respect to the GC-reaction and tumor microenvironment. The expression portraits in Figure 5a illustrate such subtype-specific activation patterns (see [4] for details). Difference portraits visualize and identify differentially expressed spots distinguishing subtypes in pairwise comparisons (Figure 5a, second row of portraits). For example, BL and DLBCL differ in the expression of spots A, B, D (BL_up: upregulated in BL) and F (DLBCL_up), while ABC- and GCB-DLBCL show differential expression of spots H (ABC_up) and A (GCB_up). Overexpression of spot module A also distinguishes DHL BCL2 versus non-DHL (DHL_up, see heatmap in Figure 5b for an overview of spot expression in the different subtypes).

Numerous gene signatures derived from differential GEP analyses have been published to distinguish BL-vs-DLBCL [3,18,30], ABC-vs-GCB DLBCL [17,31] and DHL-vs-non-DHL DLBCL [5] (Table 2, see also [22] for a review). In part, these were derived from each other. In the following, we have analyzed how these signatures perform in the MMML-data set. By that, we take into account that some of the classifiers were derived from parts of the MMML dataset like the Hummel et al. dataset [3]. Moreover, for sake of simplicity we only take into account the direction of gene expression differences (up and down) and dismiss any weighting of genes as applied for some classifiers. The published gene sets not only differ in the number of genes but do also show different degrees of mutual gene overlap. While the largest BL_up and DLBCL_up sets (Hummel et al. [3]) include the smaller ones (Sha et al. [30] and Masque-Soler et al. [18]), the two latter BL_up sets do only partly overlap (Figure 5c). Such a lack of agreement does not surprise because of “methodical noise” owing to, e.g., different pre-processing and classification methods and test data, which introduces instability of the selecting marker genes (Table 2).

The BL_up signature of Sha et al. reveals a slight bias towards proliferative gene functions as indicated by specific enrichment of signature genes in spot D compared with the other BL_up signature sets showing consistent accumulation in spot B (Figure A7 and Figure A8). The ABC_up signatures reflect a systematic bias either towards plasma cell-related (spot H, Wright et al. [31], Scott et al. [17], Figure A9) or inflammation and reactive B-cell-related transcriptional programs (Masque-Soler et al. [18]). Genes accumulating in spot A are discriminative for BL-vs-DLBCL (BL_up), ABC-vs-GCB (GCB_up) and DHL-vs-DLBCL (DHL_up) as well, which makes them ambiguous and requires careful pre-selection in terms of inclusion criteria of tumors (Figure A11). This ambiguity reflects overlapping functional and cellular programs of different lymphoma subtypes partly related to different stages of the GC reaction (Figure 5b).

We further compared the literature signatures with the spot modules containing considerably more genes (between 129 and 1154 in spot E and D, respectively, Figure 5b) than the classifiers under study (maximum 47 genes in BL_up, Table 2) and showed different degrees of overlap (Figure 5d). For BL-vs-DLBCL classification, BL_up literature classifiers and spot B perform nearly equally providing virtually equal area-under-the-curve (AUC)-values above 98% (Figure 5e). Similar results were obtained for DLBCL_up classifiers and spot F (AUC >96%), for ABC_up sets and spot H (AUC >92%) and DHL_up and spot A (AUC >82%). For GCB selection, spot A performs slightly inferior to the classifiers taken from Scott et al. and Wright et al., but similarly to that of Masque-Soler et al. (Figure 5e and Figure A10). Taken together, sets of classifier genes taken from the literature largely compare to spot-modules of co-expressed genes extracted from the multidimensional expression landscape. We show below that marker genes can be taken out from these modules in different ways without marked loss of classification power.

**Table 2:** Classifier signatures used for evaluation. An interactive classifier tool is implemented in the opoSOM browser (Appendix B and [62] for a short description).

Classifier ^(a),(f)^	Reference	Classifier Sets ^(b)^	Sample Size and Platform ^(c)^	Comment ^(d)^	Spot ^(e)^
BL-vs-DLBCL (& intermediate) ^(f)^	Hummel et al. [3]	BL_up: 47 DLBCL_up: 18	BL, DLBCL (N = 221) Array (HG U133A)	Linear model (shrunken centroids [63])	BL_UP: B DLBCL_UP: F
BL-vs-DLBCL	Sha et al. [30]	BL_up: 16 DLBCL_up: 11	BL, DLBCL (N = 1177) Array (HG U133A, lymphochip)	Support vector machine (SVM)	
BL-vs-DLBCL (& intermediate)	Masque-Soler at al. [18]	BL_up: 6 DLBCL_up: 4	BL, DLBCL (N = 90) Multiplex count (nCounter)	Linear classification	
ABC-vs-GCB (& unclassified)	Masque-Soler at al. [18]	ABC_up: 9 GCB_up: 11	DLBCL (N = 90) Multiplex count (nCounter)	Linear classification	ABC_UP: H GCB_UP: A
ABC-vs-GCB (& unclassified)	Scott et al. [17]	ABC_up: 7 GCB_up: 6	DLBCL (N = 119) Array (Nanostring)	Weighted average	
ABC-vs-GCB (& unclassified)	Wright et al. [31]	ABC_up: 13 GCB_up: 7	DLBCL (N = 274) Array (HG U133A, Lymphochip)	Linear classification ^g)^	
DHL-vs-non DHL (DHL-*BCL2*)	Ennishi et al. [5]	DHL_up: 31 Non-DHL_up: 47	GCB DLBCL (N = 157) RNA sequencing	Weighted average	DHL_UP: A n-DHL_UP: F

^(a)^: Classifier derived for two-group-comparisons, “grey zone” intermediate/unclassifiable groups are indicated, if considered. ^(b)^: Number of upregulated transcripts (Ensemble-IDs) in the classifier sets for each of the groups. For the sake of simplicity and comparison, we considered all signatures as unidirectionally upregulated (between the groups) classifier sets of genes. For distinguishing, e.g., BL-vs-DLBCL we split the list of signature genes into genes up- or downregulated in BL and subsumed them as “BL_up” and “DLBCL_up” sets, respectively. ^(c)^: Number of cases (tumor samples) and experimental platform used to derive the classifiers. The number of transcripts/genes measured varies from about 800 (nCounter multiplexing and NanoString arrays) to 15,000–20,000 (microarrays and sequencing). ^(d)^: Method used in the original publication. ^(e)^: Spot module accumulating the classifier genes. This classification used part of the MMML-data as test data, namely BL: 44 out of 74 cases; DLBCL: 176 out of 430 cases. However, raw chip data were processed completely differently here, by applying hook-calibration and standard SOM-pre- and post-processing which differ from the methods used in [3]. ^(f)^: The method uses a weighted sum of (log-)expression values as classifier. No difference between weighted and non-weighted sum was found in our data (not shown).

### 3.5. Distinguishing BL

**Figure 6:**
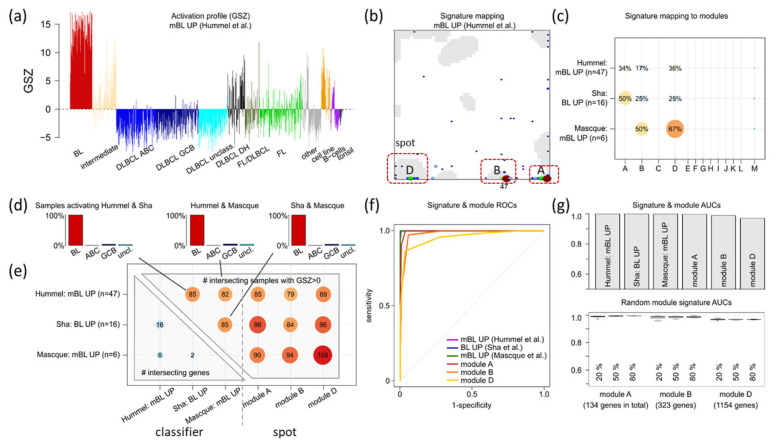
Identifying BL: (**a**) Gene set Z-score (GSZ) profile of the BL_up signature of Hummel et al. [3] along different sample strata of the MMML-data set showed activation in BL and deactivation in DLBCL (see Figure A8 for the other signatures). (**b**) Mapping of BL_up classifier genes of Hummel et al. [3] into the transcriptome landscape of lymphomas reveals their accumulation in/near spots A, B and D. Genes are shown by dots (larger dots indicate multiple occupied pixels) while spots are marked in grey. (**c**) Distribution of BL_up signature genes (rows) among spot modules (columns) indicates their unique presence in spot B and biased accumulation of Sha et al. signature genes in spot D related to proliferative cell functions. (**d**) Overlap fraction of samples of different strata found activated (GSZ >0) in pairs of BL_up classifiers which reflects 100% identification of BL and only weak (<5%) detection of “false-positive” non-BL lymphomas. (**e**) Mutual overlap matrix indicates overlapping BL_up signature genes (left lower triangle) and overlapped detection of samples with GSZ > 0 for the respective signatures and modules (right part). The signature of Masque et al. [18] and spot D detect most BL cases, however, also a relatively large number of DLBCL with proliferative characteristics (see Figure A7 and Figure A10). (**f**) ROCs of the BL_up signatures reveal only a tiny penalty for spots A and B compared with the signatures taken from literature (see Figure A10). (**g**) Area under ROC (AUC) of the curves shown in part f. AUC is virtually independent of the number of genes randomly selected from the spot modules (part below, fraction of selected genes is 20–80%, 100× resampling).

For a closer look, we applied three BL_up classifier signatures [3,18,30] to the MMML-data set. Their gene set Z (GSZ)-profiles strongly and specifically upregulate in BL (red bars) and downregulate in DLBCL (light and dark blue and cyan bars in Figure 6a for the Hummel et al. and Figure A8 for the other signatures) as expected. The signature genes accumulate in and near spots A, B and D (Figure 6b). The degree of accumulation in B is consistently high for all three BL_up signatures studied, while proliferative spot D also accumulating *MYC* targets, is slightly overrepresented for Sha et al. (Figure 6c and Figure A8).

ROC curves (Figure 6f) and AUC values (Figure 6g) are very similar for the different literature and the spot-module signatures as well (Figure 6d,e). The proliferative module D-signature performs slightly inferior because it overexpresses also in proliferative DLBCL cases. Reduction of the numbers of genes by random selection from the module sets does virtually not decrease the AUC values (Figure 6g, part below). This result implies that the number of similarly performing co-expressed genes is in the order of several dozen at minimum. In other words, there is a reservoir of suited classifier genes exceeding the number of genes included in the literature signatures.

### 3.6. Distinguishing DLBCL: ABC, GCB and DHL

**Figure 7:**
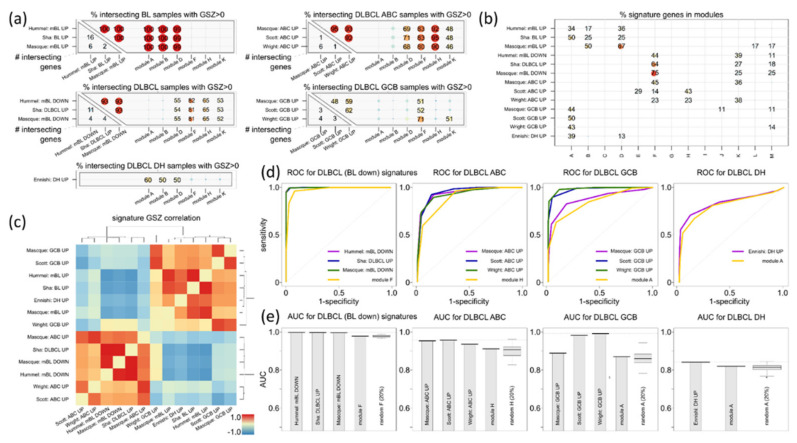
Distinguishing lymphoma subtypes: (**a**) Mutual overlap matrices of signature genes (left-lower part of the matrices) and of samples with GSZ > 0 for literature signatures and spot modules (right part of matrices) of BL_up, DLBCL_up, ABC_up, GCB_up and DHL_up reflect different discrimination power of the spots (see Figure A9 and Figure A11 for details on ABC-versus-GCB and DHL analyses). For example, “proliferation” spot D identifies BL, but also DLBCL in non-negligible amounts. Spot F, in turn, detects DLBCL but not BL. See also Figure A3 and Figure A7 for an overview. (**b**) Overlap of signature genes (rows, see vertical axis) with genes collected in the modules (columns, see horizontal axis). Spot B specifically contains only BL_up signature genes and spot H only ABC_up signature genes while most other spots (e.g., A, D, and F) un-specifically pop-up in different subtypes and thus overlap with different signatures. (**c**) The pairwise correlation maps of the GSZ-profiles of the literature classifier signatures in the MMML-data set reveal two major clusters collecting either BL_up, GCB_up and DHL_up (because of DZ resemblance of the respective subtypes) or DLBCL_up and ABC_up (LZ resemblance). (**d**) ROC characteristics compare literature with spot classifiers. (**e**) AUC values correspond to the ROC curves shown in (**d**) and to 100× resampling of random subsets of 20% of the genes taken from the spot modules (right box in each of the plots).

Next, we analyzed DLBCL_up (alias BL_down) signatures as well as classifier signatures for ABC-versus-GCB-DLBCL [16,64] and DHL-versus-nonDHL-DLBCL [5]. Overlap and distribution of tumors and of signature genes show a diverse pattern (Figure 7a,b, respectively), where BL cases specifically associate with BL_up (spot B) and ABC-DLBCL with ABC_up (spot H), while other features are more common, such as spot D upregulated in BL and in part of DLBCL due to overlapping proliferative characteristics (see above). Spot A ambiguously upregulates in BL, GCB-DLBCL and DHL, because of commonly activated transcriptional programs.

Overall, the expression values of the literature signatures under study form two major clusters Figure 7c), one being governed by dark zone functionalities (BL_up, GCB_up, DHL_up), while the other one showing partly light zone characteristics (DLBCL_up, ABC_up). The different literature signatures and the module sets show overall comparable results when applied to classify lymphomas (Figure 7d,e). Small performance differences between GCB_up and the spot A signatures can be attributed to the ambiguous functional background of this signature overlapping with that of BL_up and DHL_up and also to the fact that genes of other spot modules such as K (B-cell differentiation) activate in GCB tumors (see the map of classifier genes of Scott et al. in Figure A9). The DZ-like characteristics of the GCB_up sets should be understood as a differential feature in comparison with ABC_up (Figure 7c).

In summary, classifiers distinguishing GCB and ABC from the literature mostly perform on comparable levels in comparison with the simple “spot” sets. The signatures analyzed split roughly into two groups mainly reflecting DZ- or LZ-characteristics, because the relevant differential expression (BL-vs-DLBCL, GCB-vs-ABC) spans the DZ-LZ axis. GCB_up and DHL_up signatures partly refer to similar cell functions rendering them per se relatively unspecific requiring proper pre-selection of the cases using additional criteria.

### 3.7. Pattern Types, Microenvironmental and GC-Related Categories

**Figure 8:**
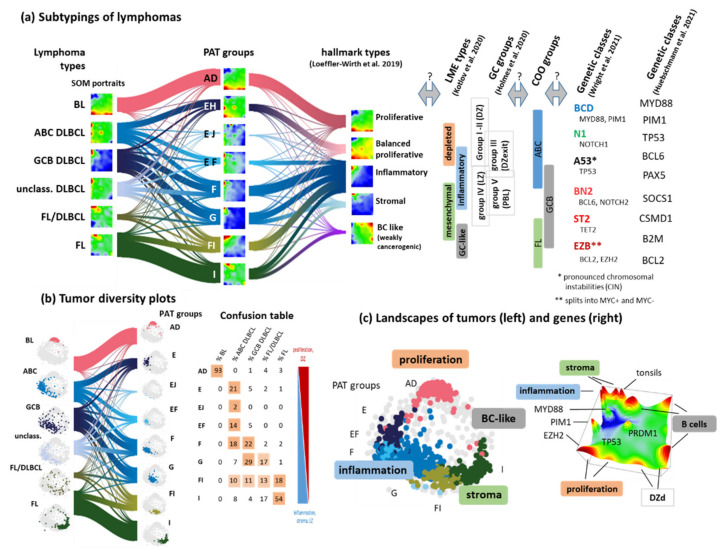
Diversity of lymphoma classification schemes. (**a**) The river plot reflects the mutual fuzziness, especially of DLBCL, between three stratification schemes, namely, lymphoma types, PATs and hallmark types [4]. PAT annotations refer to combinations of expressed spots, e.g., AD stands for the group showing typically joint appearance of spots A and D. The relation to other stratification schemes based on Lymphoma microenvironment (LME) [20], single-cell transcriptomics [24] (see assignments of groups I–V in Figure A6), cell of origin (COO) classes [16] and genetic classifiers using WES [13] or WGS [65] is partly not clear and needs consenting. (**b**) Systematic changing spot patterns in the expression portrait in part (**a**) associate with systematically changing tumor patterns in sample state space (each dot refers to one sample). The confusion table quantifies the mutual overlap and illustrates their systematic shift (colored background). (**c**) Different regions of both, sample (left part) and gene expression (right part) state space assign to different lymphoma types and hallmark characteristics. Position of key mutated genes is indicated.

Transcriptomic portraits of lymphoma subtypes show “fingerprint”-like spot patterns which typically include more than one spot module (Figure 8a). We recently proposed a hierarchy of lymphoma strata of increasing granularity making use of topological features of the gene expression landscape in terms of so-called pattern types (PAT). PATs are defined as combinations of activated expressed spot-modules [4] (Figure 8a,b, left part). The majority of BL and FL assign virtually to one PAT-group, each in an almost one-to-one relation. In contrast, DLBCL and FL/DLBCL reveal a strong mixing among five to six PAT-groups, thus reflecting the high molecular diversity of these lymphoma types. There is also pronounced enrichment of ABC-, GCB- and FL/DLBCL tumors in the direction from proliferative DZ-related towards inflammatory/stromal LZ-like characteristics. The PAT-groups and the underlying order of spot activation thus follow roughly the course of the GC-reaction discussed above. The single-cell states suggested by Holmes et al. and the derived prognostic COO-groups I–V [24] order similarly (Figure 8a, right part). Another recent study classified DLBCL into four lymphoma microenvironment (LME) categories of proliferative (depleted of bystander cells), mesenchymal (enrichment of stromal cells and extracellular pathways), inflammatory (inflammatory bystander cells) and GC-like (enriched in GC-cells) characteristics [20], which notably agree with the functional background of the major spot modules (Figure 8c) as well as in the immune cell composition of the PATs [4]. For example, spot D in our landscape associates with DZ characteristics (group I, see [24]) and proliferative lymphoma types depleted from bystander cells [20], while spot F is representative for LZ-like (group III) and inflammatory LME lymphomas (Figure 8c). Interestingly, the four LME-types reveal correspondence with “hallmark” types of lymphomas related to “proliferative”, inflammatory, balanced inflammatory (stromal) and weakly cancerogenic (B-cell like) characteristics extracted from the PAT groups [4] (Figure 8a). Hence, diversification of subtypes using combinations of expression modules, single cell-derived COO or LME categories provides new insights about functional aspects of the tumors with impact for the selectivity of classification schemes.

## 4. Discussion

### 4.1. Footprints of the GC Reaction in the Lymphoma Transcriptome

SOM portrayal provided a holistic view on the transcriptome landscape of lymphomas and its multidimensional nature. It appears as a network of cellular programs, each governed by the transcription of a few dozen to a few hundred co-regulated genes of different functional context, related to, e.g., aberrant GC-biology, B-cell maturation and/or tumor microenvironment. Recent single cell expression signatures refined details of the GC reaction and enabled one to identify cell-of-origin footprints in the lymphoma transcriptome with impact for its subtyping, particularly as rationale for its continuous and multidimensional nature. The SOM landscape of lymphomas reflects the time-course of the GC reaction as series of transcriptional states from DZ via intermediate towards LZ and PBL. As for lymphomas, also the transcriptional states along the GC reaction form a continuum on cellular resolution rather than a set of well-separated single cell states [46]. Notably, SOM-landscapes in general are able to recapitulate time-dependent trajectories along such a continuum in gene state space [66] as was shown for a wide spectrum of time-scales ranging from cell and tissue development [67] to evolutionary processes [68,69] using transcriptomic and genetic features.

We looked at different lymphoma classes in the light of this complex transcriptional landscape and considered novel single-cell transcriptomics data which imply parallels between transcriptional programs in the GC and different lymphoma types. Knowledge emerging from transcriptomic studies increasingly identify genomic mechanisms driving lymphomagenesis towards different tumor phenotypes. In addition to the basic types BL, DLBCL and FL, we characterized provisional intermediate entities with specific genetic lesions affecting driver genes of lymphoma genesis, namely, double hits of *MYC* and *BCL2* combining genetic features characteristic for BL and FL, of rearranged *IRF4*, a key player of plasma cell activation, as well as Chr. 11, giving rise to a BL-resembling transcriptome: however, via an alternative pathway. All these classes occupy intermediate positions in the transcriptomic landscape, thus partly providing a genetic reasoning of their phenotypes. Our transcriptomic characterization supports the new, 2022 adaptation of WHO nomenclature of lymphoid tumors regarding these classes where *MYC/BCL6* rearrangements are not further considered as a hit-classifier and the other two types are now defined as IRF4-R LBCL and HGBL-11q, respectively (Table 1).

### 4.2. Navigating a Multidimensional Transcriptional Landscape

The continuous character of the transcriptome landscape not only complicates subtyping but also definition of gene expression classifiers. We find that existing classifier signatures developed for two-class comparisons in different studies are often redundant, i.e., they perform similarly regarding sensitivity and specificity even if the respective sets of marker genes only weakly overlap. Our analysis revealed two major causes of “redundancy”: firstly, “statistical-selection uncertainties”, due to the reservoir of correlated genes in the relevant modules exceeding the number of marker genes usually selected; and secondly, “biological uncertainties”, due to continuously distributed transcriptional states resulting in overlapping subtypes in a complex molecular landscape. We demonstrated that classifier signatures extracted from modules of this landscape performed as well as classifier signatures derived from pairwise comparisons of pre-selected subtypes. This result is not surprising, because both concepts use the same data, however, in a different order, namely, by selecting classes of interest in the data before or after bioinformatics diversity analysis, respectively.

Overall, it was not our aim to design another, “better”, classifier but simply to compare modules of co-regulated genes obtained by means of multidimensional clustering with the classifiers specifically designed for two-group classification tasks taken from the literature. We envisage an advantage of the holistic approach due to the fact that it better resolves the whole network of relevant cellular programs, and thus it enables the joint control of the “statistical” and “biological” redundancy problems. For example, the plasma cell characteristics in our landscape are consolidated by the consideration of plasma cell myeloma cases strongly expressing the plasma cell signature, which, in turn, appeared to be an essential characteristic of ABC-DLBCL (spot module H). In addition, inclusion of FL helped to better resolve FL/DLBCL and DHL, partly expressing joint characteristics of GCB-DLBCL, BL and FL (spot module A). Finally, sorted reactive B-cell specimens and tonsils extended the data space towards the GC B-cell and lymph node signatures and, particularly, enabled one to identify “outlier” samples strongly contaminated with reactive cells in a previous study [19]. Overall, the holistic approach allows to assemble the lymphoma transcriptome in terms of modular building blocks of concertedly expressed genes with underlying functional features. This view helps to stratify lymphomas in terms of combinatorial PATs and to interpret smaller subclasses in the context of larger ones.

### 4.3. Consenting Classifiers as a Trade-Off Balancing Accuracy and Stability

**Figure 9:**
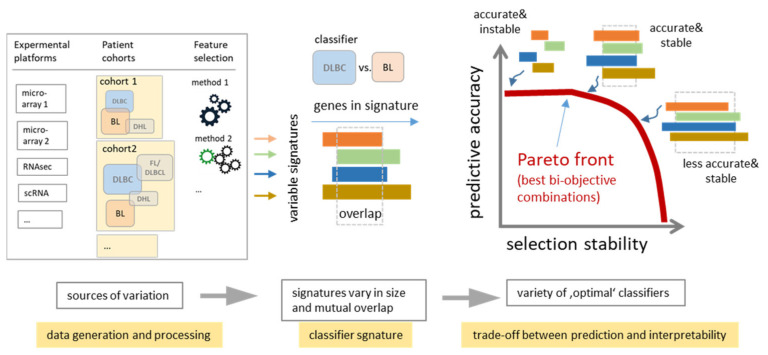
Extracting classifiers as a trade-off between prediction accuracy and stability classifiers for the same classification task (e.g., BL versus DLBCL) can vary owing to many reasons (e.g., measurement platforms, size and composition of test cohorts, data processing methods) resulting in “instability” of marker signatures due to varying size and composition. Bi-objective optimization between prediction accuracy and stability can be understood as “Pareto”-front representing a manifold of optimal combinations along a curve indicating that considerable gain in stability (and thus consent between the signatures supporting their interpretability) can be achieved virtually without loss of prediction accuracy (the schematic view is adapted from [71]).

Molecular subtyping requires a set of bioinformatics tasks such as standardized preprocessing, cluster analysis (finding and defining classes), supervised classification (assigning cases to the classes) and classifier building (selecting features discriminating the classes) as well as molecular biological and clinical characterization of the classes [70]. For each of these tasks, numerous methods are available. Each of them associates with a certain level of noise, giving rise to uncertainties in accomplishing classification tasks regarding the diagnostic markers selected and the associated functions and predicted outcomes. Classifier selection typically tries to find minimum-sized signatures providing a maximum predictive accuracy (for example, the nearest shrunken centroid method [63] applied in [3] for BL versus non-BL discrimination (Table 2). Larger-sized signature sets are often considered as “overfitted”, a view, which underestimates the stability (i.e., consistency) of selected genes and thus their biological interpretability. Hence, the intention of shrinking the size of classifiers is opposed by the need for its increase for better interpretability. A more stable feature selection could be preferential over a less stable but slightly more accurate one. In a general sense, this problem can be understood as a trade-off between predictive accuracy and selection stability (Figure 9, see also [71]) where the accuracy relates to the statistical relevance of the selected features while stability is linked to their biological interpretability and consent application. It can be considered as a Pareto bi-optimal trajectory in accuracy versus stability coordinates from which a particular solution can be selected as a compromise between both aspects according to researchers’ needs [71]. Our results show that stability gains can be reached at small cost of classification accuracy by means of our spot-module selection. It provided larger-sized signatures than the minimum-sized classifiers taken from literature at virtually similar AUC values but higher stability regarding gene overlap and interpretability.

### 4.4. New Concepts: PATs, Machine Learning of Molecular Portraits and Multiomics Classifiers

**Figure A2:**
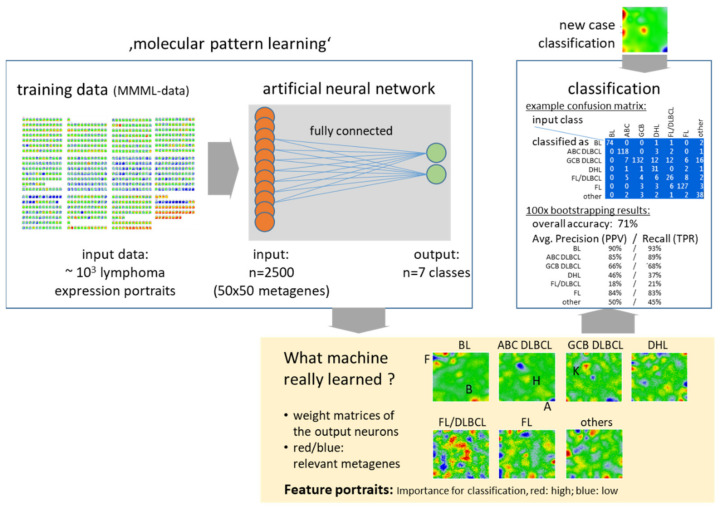
Machine learning of lymphoma subtype expression patterns: Based on expression portraits of the MMML data, the machine learned (fully connected, two-layered neural network) the expression patterns of seven subtypes in order to classify new cases. Relevant features for classification patterns are shown in the yellow box. It applied the layer-wise relevance propagation (LRP) method to visualize in SOM space what the machine really learned [84]. Classification quality was estimated as confusion matrix (input versus learned class), and precision (correct percentage of positive calls) and recall (percentage of actual true calls) values. Bootstrapping (10% randomly sampled for classification; 1000 iterations) was used for estimating the performance of classification.

**Figure A3:**
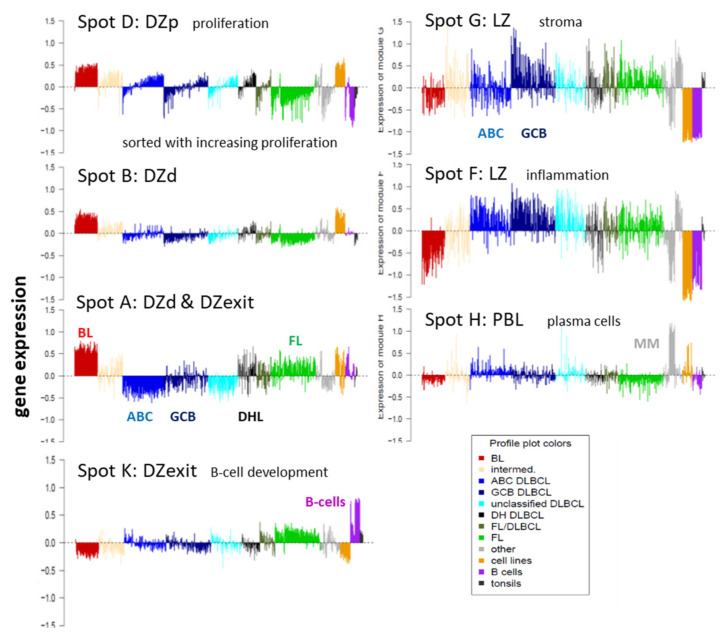
Gene expression spot profiles across the lymphoma subtypes and their association with steps of the GC reaction (see also Figure 2). Tumors are ranked with increasing cell cycle activity for each subtype using the signature of [85]. DLBCL tumors show high variability with the cell cycle while BL do not. Spot G (and F) reflects anticorrelation with cell cycle activity. Notably, spot activation follows the GC trajectory shown in Figure 2d. Spots D, B and A are upregulated in BL where D and A also activate in DLBCL and FL, respectively; spot K in FL, spot G in GCB-DLBCL, spot F in DLBCL and FL and spot H in MM (multiple myelomas) and ABC-DLBCL.

**Figure A4:**
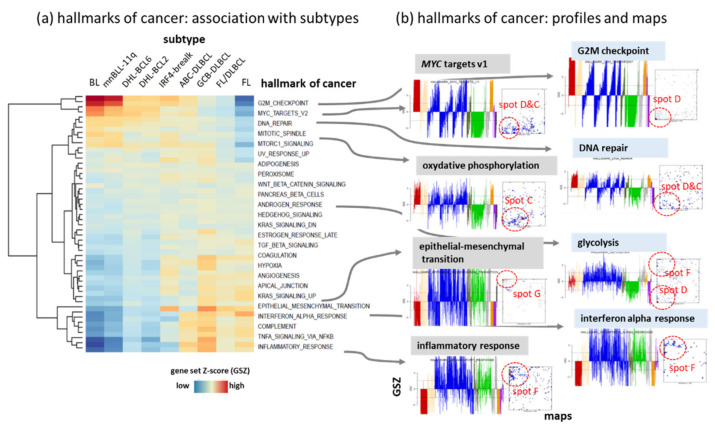
Functional associations of the subtypes and of the spot modules with gene signatures taken from the category “hallmarks of cancer” [86]. (**a**) BL and BL-resembling subtypes show high expression of functions related to cell cycle activity, DNA-repair and *MYC*-targets, while inflammatory and stromal characteristic are upregulated in DLBCL and, partly, FL. (**b**) The profiles reveal a more detailed pattern. Not that the tumors in each subtype are ranked with increasing cell cycle activity (using the literature gene set [85]) resulting partly in “ramp-like” silhouettes which reflect variance of expression as a result of changing cell cycle activity. The gene set maps show the distribution of the signature genes in the SOM. Theirs accumulation in around the spot-areas is indicated by red ellipses.

**Figure A5:**
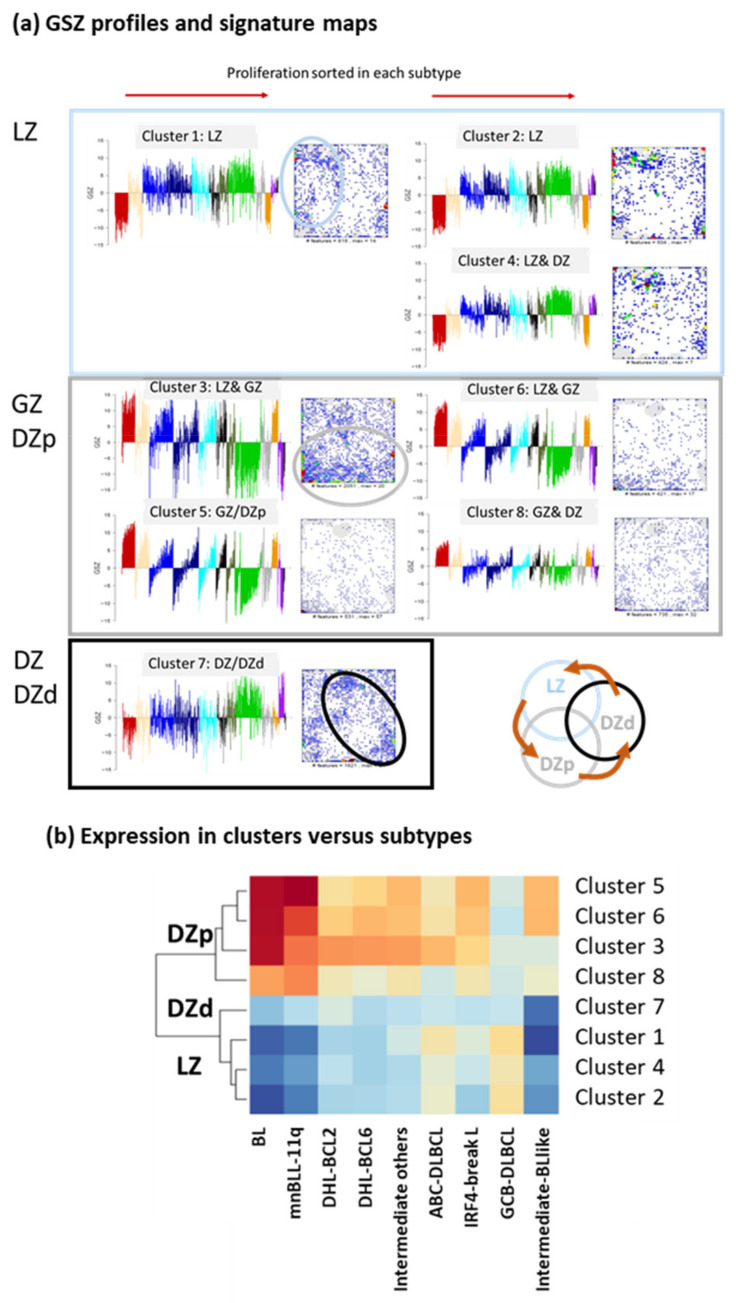
Mapping B-cell states in the GC as identified by Kennedy et al. [25] into the lymphoma transcriptome landscape: (**a**) Kennedy et al. identified 8 gene clusters which distribute over the GC states LZ, DZp (alias GZ, grey zone) and DZd as indicated in the figure. The clusters contain between 2887 (cluster 3) and 694 (cluster 4) genes (see [25]). The profiles reveal that DZp clusters are governed by cell cycle activity (tumors are ranked with increasing proliferation in each of the subtype), while LZ show higher activity in DLBC. The gene maps reveal accumulation of genes in different areas of the map as indicated by the circles which combine into the GC “circle” as visualized in Figure 2. (**b**) The heatmap shows the mean expression in the different subtypes and clusters. Hierarchical clustering splits DZp, DZd and LZ in a vertical direction, where DZp reflects BL-resemblance, LZ reflects DLBCL resemblance and DZd shows activation in BCL2 DHL.

**Figure A6:**
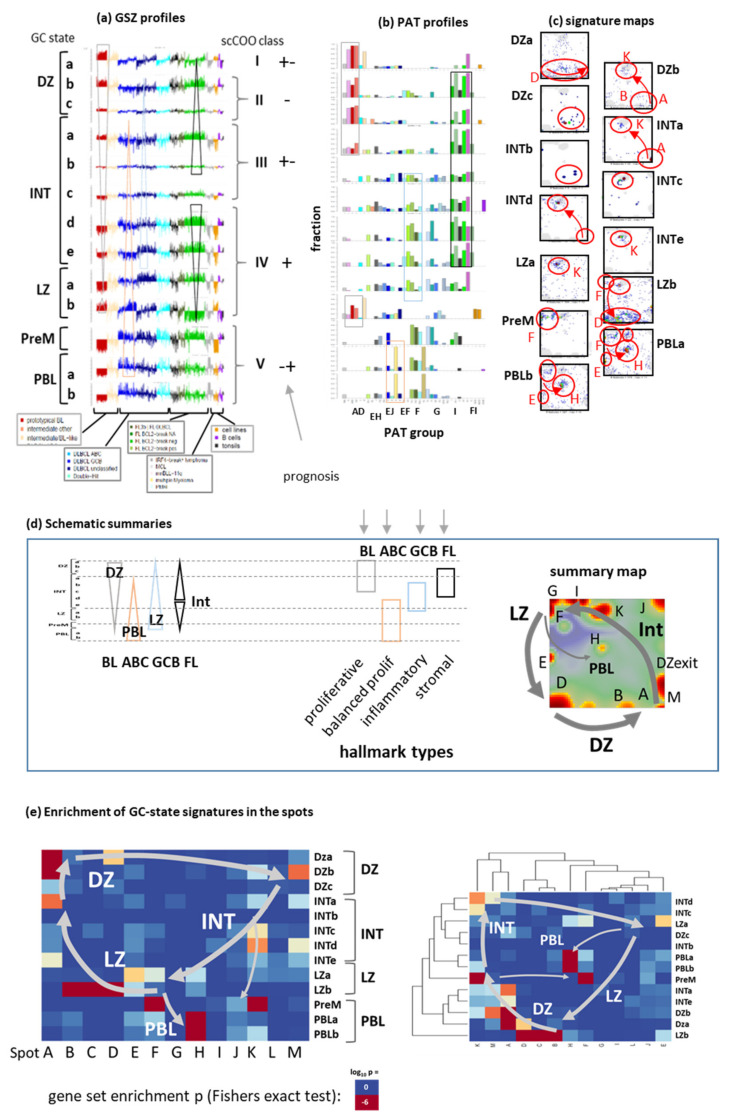
Mapping B-cell states in the GC as identified by Holmes et al. in [24] into the lymphoma transcriptome landscape: (**a**) GSZ profiles across the lymphoma reveals subtype specific up and downregulation, where upregulation in BL, ABC, GCB and FL associates with DZ, PBL, LZ and Int, respectively. Note that LZb upregulates in BL, ABC, GCB and DHL as well. scGC states were accumulated in scCOO prognostic classes I–V with inferior (−) via intermediate (−+, +−) to good (+) prognosis (see [24] for details). (**b**) Pattern type profiles in units of the fraction of the PAT per GC state. (**c**) Maps of the signature genes of the 13 GC states. Areas of accumulation are shown by ellipses. (**d**) Schematic summaries of (**a**–**c**) The triangles symbolize changing activities in the respective subtypes (left part). The PATs were summarized into four hallmark types with largest fractions in the specific subtypes as indicated. The summary map visualizes the GC trajectory in the lymphoma SOM. Letters assign relevant spots. (**e**) Enrichment of the GC-state gene signatures among the spot-modules of the transcriptomic landscape. Supervised (unclustered) and two-way clustered heatmaps of −logp (Fishers exact test) enrichment values. The GC-circle is indicated by arrows. It transforms into the picture shown in the SOM landscape in part (**d**).

**Figure A7:**
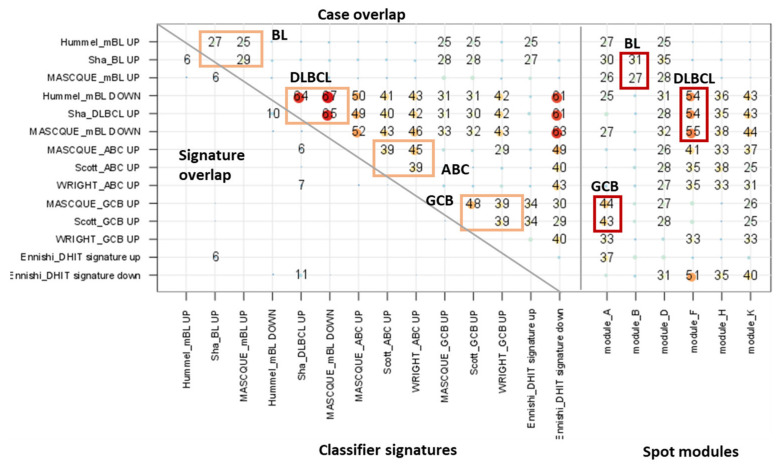
Signature gene intersection (left, lower triangle) and % jointly activated samples (GSZ >0, right, upper triangle). Only classifiers designed for the same subtypes are compared.

**Figure A8:**
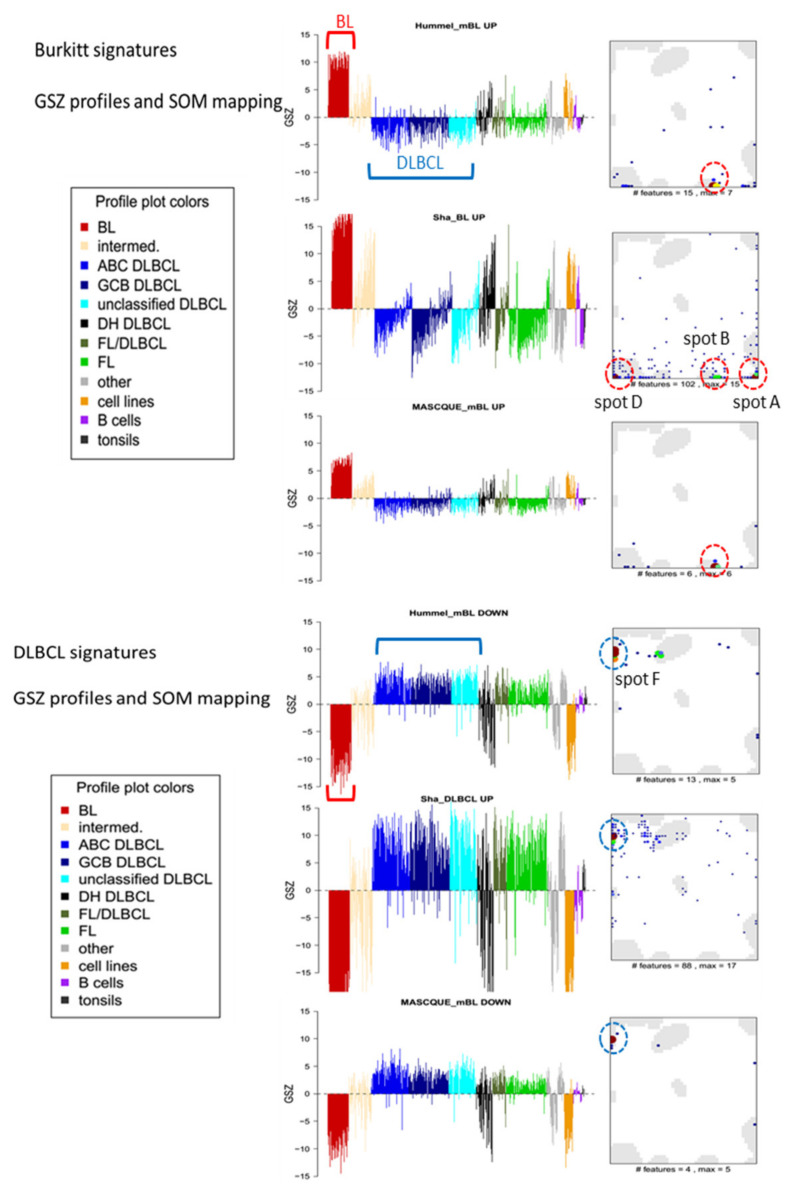
Characteristics of BL-vs-DLBCL signatures: GSZ-profiles of the BL_up signatures show high (GSZ >0) values for BL (red), lymphoma-cell lines (apricot) and, to a lesser degree, intermediate lymphoma (beige) and low values (GSZ <0) for DLBCL (ABC, GCB, and unclassified; blue and cyan, respectively), and FL (green). The patterns virtually reverse for DLBCL_up signatures. SOM mapping indicates accumulation of signature genes, first of all, in/near spot B but also in/near spots D and A (red circles). Especially, part of the Sha-BL_up signature genes are found in spot D which associates with increased cell division. Samples in each subtype were sorted with increasing expression of the cell-cycle activity from the left to the right. The increasing slope of the Sha-BL_up GSZ-values along most of the subtypes reflects the relation of this signature to proliferation.

**Figure A9:**
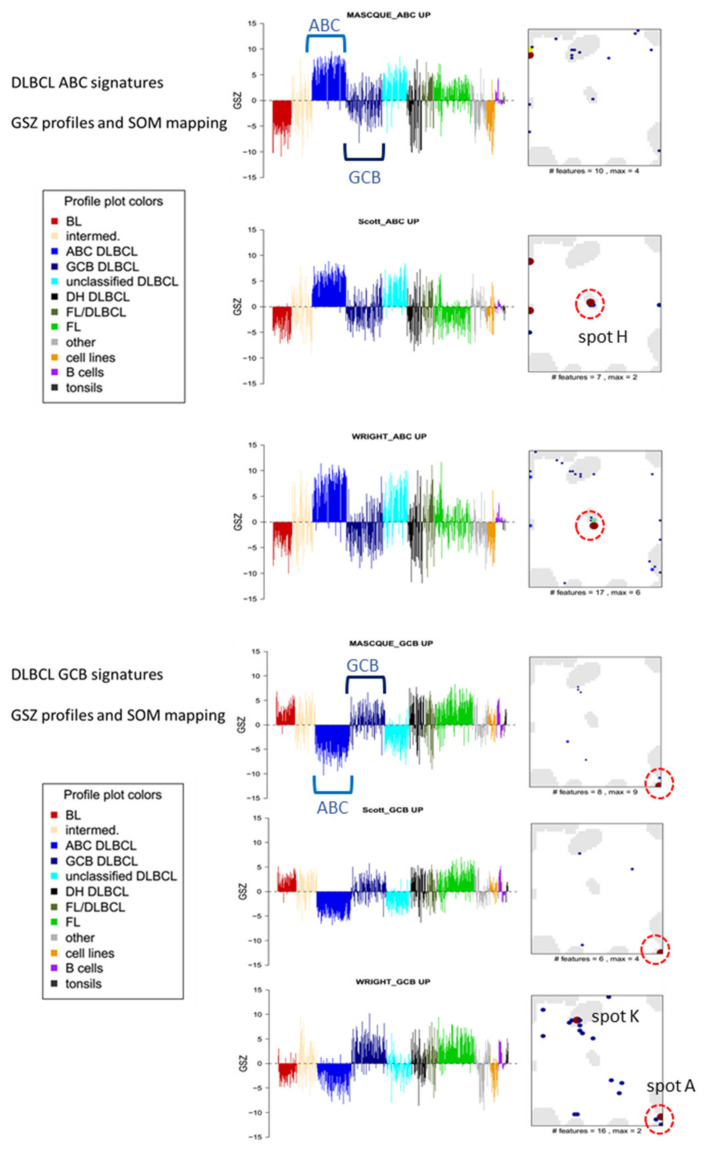
Characteristics of ABC-vs-GCB DLBCL signatures: GSZ-profiles of the ABC_up signatures show high (GSZ >0) values for ABC-DLBCL (light blue) and unclassified DLBCL (cyan) and low values (GSZ <0) for GCB-DLBCL (dark blue) and BL (red). SOM mapping indicates accumulation of signature genes, first of all, in/near spot H but also in/near spots K and F (red circles). Especially, part of the Masque-ABC_up signature genes are found in spots F/K which associate with stroma/inflammation and healthy B-cell functions, respectively, giving rise to upregulation in FL. In contrast, Scott-ABC_up and Wright-ABC_up signatures accumulate in spot H, which associates with deactivated expression in FL. The GCB_up set of Masque et al. virtually contains only genes from spot A, which explains their similar performance.

**Figure A10:**
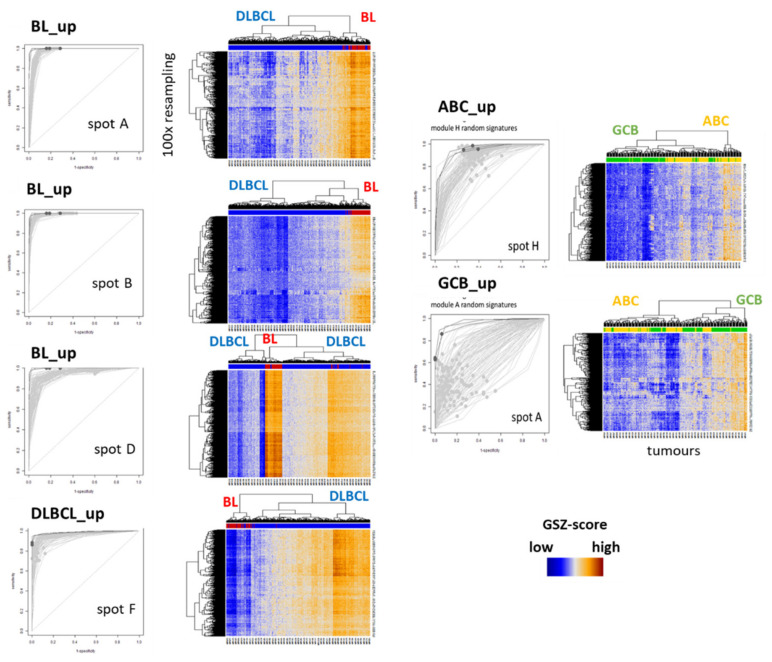
Classification of lymphoma subtypes using randomly sampled genes from the spots: For each spot module, 20% of genes were sampled randomly (100× resampling) and used as expression signature for classifying BL versus DLBCL (left part) and ABC versus GCB DLBCL (right part). The figure shows the ROC curves as well as two-way clustered GSZ heatmaps. AUC values were shown in Figure 7e. Spot B best separates BL versus DLBCL (BL-up signature) virtually independent of the selected genes. Spot D (proliferation) performs (BL-up) worse because also part of DLBCL show increased GSZ-values. The ABC-versus-GCB classification (right part), especially GCB_up (spot A), shows large variability of classification power. ROC curves were obtained by shifting the classification threshold from minimum to maximum GSZ-values. The dots along the curves refer to a threshold at GSZ = 0.

**Figure A11:**
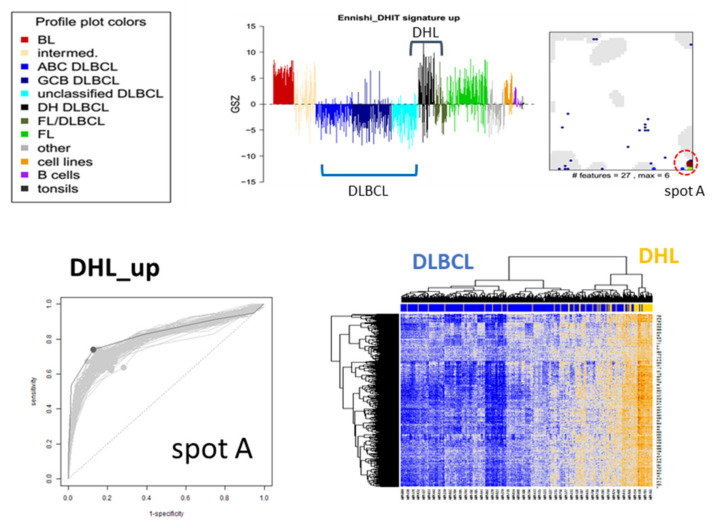
Characteristics of the DHL signature [5]. Note that this signature was designed for distinguishing DHL and DLBCL. Other subtypes such as BL and FL are shown here for illustration, particularly to show the joint BL and FL resemblance of DHL. The part below shows the resampling of genes from spot A as described in the legend of Figure A10.

In a practical sense, the Pareto concept discussed in the previous subsection intends increased, more stable marker signatures instead of the minimalistic sets often used. One option to achieve this would be using genes included in the spot modules extracted from multidimensional analyses such as SOM or non-negative matrix factorization methods [72] as classifier signatures. However, selection of single modules as marker sets can be insufficient due to the ambiguous nature of most of the spot modules; for example, spot A is up in BL, FL, DHL and GCB while spot D is up in BL and DLBCL as well. The concept of patterns types (PAT) applied previously [4] refines classification by using combinatorial patterns of more than one activated spot-modules: e.g., the ambiguity of spot A partly resolves because in parallel either spot D or G can be found activated assigning the tumors alternatively as BL (or DH) or FL, respectively (Figure 1e). However, in practice, PATs do not completely resolve the uncertainty of subtype classification mainly because of the continuous character of the transcriptomic landscape (Figure 8a). The next conceptual step to increase granularity of classifier selection consists of considering the whole transcriptome portraits of the tumors with metagene resolution. For a proof of principle, we applied fully connected neuronal network machine learning for molecular portrait recognition in analogy with face recognition in security identification applications (Appendix A.4, Figure A2). The method is trained by the class labels of the lymphoma portraits and returns class prediction of newly added lymphoma portraits. We find that the machine indeed learns relevant features beyond the spots and, overall, can be seen as an option to exploit maximum information content of the transcriptome landscape. Its limitation, on the other hand, is the averaging inherent in the metagene patterns as well as the lack of clear-cut borderlines between the transcriptomes of the subtypes, which possibly limits its resolution by principle reasons.

One way to overcome this problem can be seen in using multi-omics molecular information and particularly, genetic data as another way to improve classification. For example, subtyping of gliomas has been based mostly on a series of genetic markers [73]. For lymphomas, the genetic patterns look overall more complicated. However, well-defined genetic lesions such as double hits, *IRF4*-rearrangements and aberrant Chr. 11q are now considered as genetic markers for specific lymphoma sub-entities [32]. Transcriptomics enabled us to interpret their functional phenotypes in the transcriptomic landscape of lymphomas. On a wider scale, genetic data of lymphomas become increasingly available and provided novel insights about genetic drivers [11,12,74], particularly of BL [75], FL [54] and DLBCL [11,12,74] (see also [14,76] for a review). Seven genetic DLBCL subtypes with distinct outcomes were extracted using whole-exome sequencing (WES) [13] (Figure 8a, right part). They revealed a similar shift of molecular function as the transcriptomic classes, and particularly, our PAT groups. A recent whole-genome sequencing (WGS) study [65] distinguished nine subtypes partly resembling the WES-based ones (Figure 8a). It was found that B-cell lymphomas can be traced back to mutated cells that emerge from the GC and/or have passed the GC reaction. This result rationalizes the parallels between the cellular states of the GC reaction and lymphoma transcriptomes discussed. We expect that genetic classification schemes will extend and refine the molecular subtyping of lymphomas. Integrative views and bioinformatics methods linking them with the gene expression landscape as well as with epigenetic, proteomic and metabolomic data are, however, required for a holistic understanding of factors governing the heterogeneity of lymphomas.

## 5. Conclusions

The whole-transcriptome SOM landscape of GC-derived lymphomas in combination with portrayal of the individual tumors offers novel perspectives for consenting existing redundant classifier signatures, for their better functional understanding in the light of the GC reaction and for increasing granularity of classification towards personalized diagnostics. Consent between different gene expression signatures as well as their extension towards existing novel classification schemes such as PAT grouping, hallmark types, LME groups and single cell-derived COO classes, are needed to combine their different functional perspectives into a common view on the molecular phenotypes of lymphoma. The spot-modules of co-regulated signature genes represent “mountain peaks” used as classification landmarks in an otherwise continuous transcriptomic landscape of GC-derived lymphomas, which leaves classification uncertain to some residual degree. Still existing inconsistencies between genetic, transcriptomic and microenvironmental classifiers need further research with larger tumor cohorts to better resolve rare classes. Improved experimental techniques such as single-cell omics measurements on the data side and probabilistic classification schemes and integrative multi-omics bioinformatics in combination with machine learning on the analytics side are the next milestones along the avenue towards further improved, classification of lymphomas.

## Figures and Tables

**Table 1:** New nomenclature for genetic entities considered by WHO 2022 [32].

Previous Nomenclature (This Paper)	New Nomenclature (WHO 2022)	Comment
DHL MYC + BCL2	DLBCL/HGBL MYC/BCL2	
THL MYC + BCL2 + BCL6	DLBCL/HGBL MYC/BCL2 and BCL6	BCL6 rearrangement is not considered as “hit“ defining an entity
DHL MYC + BCL6	DLBCL or HGBL, NOS	
MYC-negative Burkitt-like lymphomas with Chr. 11q aberration pattern (mnBLL-11q)	High-grade B-cell lymphoma with 11q aberration (HGBL-11q)	Reference to BL is removed
IRF4-rearranged large cell lymphoma (IRF4-LCL)	Large B-cell lymphoma with IRF4 rearrangement	

**Figure A1:**
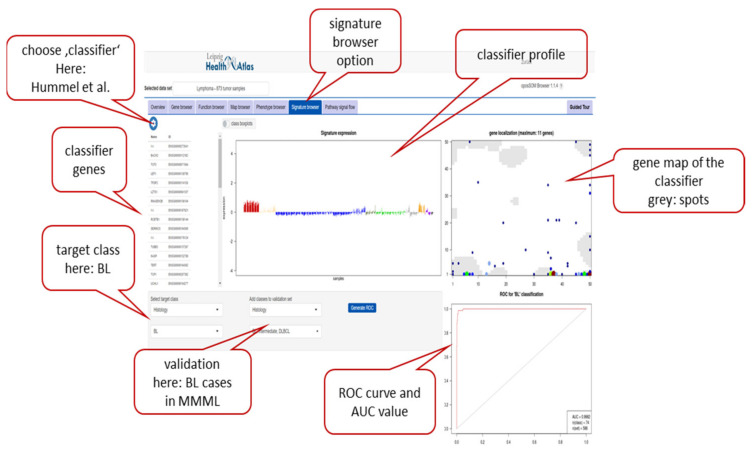
Screenshot of the signature browser implemented in the opoSOM browser.

## Data Availability

An interactive online browser is provided (Appendix A.3, and Figure A1), which provides different views on the MMML data set including the evaluation of gene-marker sets.

## Glossary

ABC	activated B-cell-like subgroup
AUC	area under the (ROC) curve
BL	Burkitt lymphoma
Chr	chromosome
COO	cell of origin
DHL	double-hit lymphoma
DZ	dark zone of germinal centre
DZd	cell state of differentiation in dark zone of germinal centre
DZexit	B-cells that just exit the dark zone of germinal centre
DZp	cell state of proliferation in dark zone of germinal centre
DLBCL	diffuse large B-cell lymphoma
DNA	deoxyribonucleic acid
FL	follicular lymphoma
GC	germinal center
GCB	germinal center B-cell-like subgroup
GEP	gene expression profiling
GSZ	gene set Z-score
HGBL-DH/TH	high-grade B-cell lymphomas with double or triple hits
HGBL-NOS	high-grade B-cell lymphomas, not otherwise specified
LCL	large cell lymphoma
LME	lymphoma microenvironment
LRP	layer-wise relevance propagation
LZ	light zone of germinal center
MCL	mantle cell lymphoma
mnBLL-11q	*MYC*-negative Burkitt-like lymphoma with 11q aberration
MM	multiple myeloma
MMML	‘Molecular Mechanisms of Malignant Lymphoma’ consortium
mRNA	messenger ribonucleic acid (RNA)
PAT	pattern types
PBL	plasmablast
PMBL	primary mediastinal B-cell lymphoma
PrePC	precursors of plasma cells
PreM	precursors of memory B-cells
ROC	receiver operator curve
SHL	single hit lymphoma
SOM	self-organizing map
THL	triple hit lymphoma
WES	whole-exome sequencing
WGS	whole-genome sequencing
WHO	World Health Organization

## Appendix A. Additional Methods

### Appendix A.1. Transcriptome Map of Lymphoma

We here reanalyzed the data set collected in the frame of the MMML (Molecular Mechanisms of Malignant Lymphoma) project (see [4] and references cited therein). The lymphoma samples divide into ten major strata based on pathological evaluation, genetic and/or previous gene expression classification criteria listed in the Materials and Methods section. Details of the generation of the transcriptome map of lymphomas were described previously [4].

### Appendix A.2. GSZ Profiles of Classifier Signatures and ROC Characteristics

Each (reference or spot) classifier comprises a set of genes (alias signature). It provides one GSZ (gene set Z-score)-value per lymphoma case. The GSZ-score is defined as the mean centralized log-expression (with respect to mean log-expression averaged over all samples) in each sample averaged over all genes of the set and divided by its standard deviation [77]. Absolute GSZ-values are large in case of large differential expression and small variance of the set genes. GSZ-values were calculated for reference signatures and spot signatures as well.

We classified samples with GSZ-values greater than zero as “selected” by the respective classifier signature. For example, all samples with GSZ >0 of the BL_up signature were classified as BL, and samples with GSZ >0 for DLBCL_up were classified as DLBCL. For comparing reference with spot signatures, we counted the number of overlap samples showing values of GSZ >0 for both classifier signatures. Receiver operator curves (ROC) were generated by shifting the classification threshold value between the minimum and maximum GSZ-values of the respective signature and using the class-assignment of samples in the MMML-cohort as gold standard. Area under the curve (AUC) was calculated as integral of the respective ROC curve.

### Appendix A.3. oposSOM Browser of the MMML Lymphoma Data Set

We provide the so-called oposSOM browser for the MMML data set available on the Internet at https://apps.health-atlas.de/opossom-browser (accessed on 3 June 2022). It enables one to discover the transcriptome map of B-cell lymphoma under different perspectives (see [62] for a short description of the browser). The “Signature browser” option (click the respective button) enables to interactively discover the classifier signatures used in this publication, and also custom ones as lists of genes entered by the user. Particularly, one can select (i) a signature set, either predefined (i.e., taken from Table 2) or a custom one by copying a list of genes into the respective field, (ii) a target class for the classifier signature (e.g., BL) and (iii) the subtypes chosen for validation. The browser then generates the GSZ profile and gene map of the signature set and the respective ROC and AUC data.

### Appendix A.4. Machine Learning of Transcriptonal Portraits

SOM provides transcriptional portraits for each tumor with a 50 × 50 metagene resolution. In addition to strongly protruding spot regions, they include also weakly differentially expressed areas. As such, these whole-transcriptome patterns imply classification via molecular portrait recognition similarly to face recognition in security identification or pattern recognition in imaging-based diagnostics such as computer tomography [78], diagnostics of photographic tumor images [79] or microscopic tissue slides [80] applied to lymphomas [81,82,83]. For a proof of principle of this idea, we trained a neural network to the expression portraits of the MMML data set for classification of seven lymphoma types (Figure A2). BL were superiorly identified (recall of 93%) followed by ABC-DLBCL (89%), FL (83%) and GCB-DLBCL (68%), reflecting overall good performance of the learning system. Inferior performance for DHL (37%) and FL/DLBCL (21%) is not surprising because of the composite character of their expression landscape merging features of FL with BL and/or with DLBCL. Machine learning makes use of the whole transcriptome landscape and not only of most prominent differential features, however, in terms of a “black box” without explicit annotation of the feature patterns the machine really learned for classification. The so-called layer-wise relevance propagation (LRP) method visualizes the metagene areas of high relevance for classification in SOM space [84] (Figure A2, feature portraits). On a rough scale, the relevant features agree with the differential spots discussed above, e.g., spot B and F distinguish BL versus DLBCL, and spot H and K distinguish ABC versus GCB-DLBCL. On a finer scale, the feature portraits are more granular than the difference portraits (compare Figure A2 with Figure 5a) which indicates that machine learning used relevant features beyond the spots with higher granularity. For example, genes related to B-cell activation near spot K upregulate specifically in GCB- compared with ABC-DLBCL and metagenes around spot F reveal diverse relevant patterns if BL is compared with DHL. Hence, machine learning offers the option to classify new tumor specimens based on their transcriptomic portraits, i.e., to “recognize” individual lymphoma portraits rendering the method suitable for personalized diagnostics.
